# Three-dimensional plasmonic nanopores for DNA-PAINT and dual-material Au/Si architectures

**DOI:** 10.1186/s12951-026-04509-9

**Published:** 2026-05-08

**Authors:** German Lanzavecchia, Anastasiia Sapunova, Alan M. Szalai, Shukun Weng, Ali Douaki, Makusu Tsutsui, Roman Krahne, Guillermo Acuna, Denis Garoli

**Affiliations:** 1 Istituto Italiano di Tecnologia, Optoelectronics, Genova, 16163 Italy; 2 Dipartimento di Scienze e Metodi dell’Ingegneria, Università degli Studi di Modena e Reggio Emilia, Reggio Emilia, 43122 Italy; 3 Università degli Studi di Milano-Bicocca, Milano, 20126 Italy; 4 Department of Physics, University of Fribourg, Fribourg, CH-1700 Switzerland; 5 SANKEN, The University of Osaka, 495 Osaka, Ibaraki, 567-0047 Japan

**Keywords:** Plasmonic nanopores, DNA-PAINT, Single-molecule fluorescence, Near-field enhancement, Fluorescence quenching, DNA nanorulers, Dual-material nanopores, Hybrid nanopores, Biosensing

## Abstract

Plasmonic nanopores combine nanofluidic confinement with electromagnetic field enhancement, enabling optical interrogation of single molecules in sub-wavelength volumes. Building on our previously developed three-dimensional dielectric nanopore platform, here we introduce 3D plasmonic nanopores as an optically active extension for single-molecule studies. We demonstrate, to our knowledge, the first implementation of DNA-PAINT within a solid-state nanopore, showing that transient hybridization events can be optically resolved within a confined plasmonic geometry. In parallel, DNA spacers of controlled length are used to position fluorophores at defined nominal distances from the metallic surface, revealing a non-monotonic fluorescence response consistent with the interplay between electromagnetic enhancement and metal-induced quenching, with an optimum around 6 nm. Finally, we introduce a novel dual-material Au/Si nanopore geometry as a hybrid plasmonic-semiconducting architecture. Static fluorescence measurements with DNA spacers and Rhodamine 6G reveal an optical response distinct from that of fully metallic Au nanopores, while simulations indicate asymmetric electromagnetic field distributions in the multi-material design. Together, these results establish 3D plasmonic nanopores as a platform for single-molecule optical techniques and show that dual-material nanopores provide an additional design parameter for modifying nanoscale optical response.

**Graphical Abstract:**

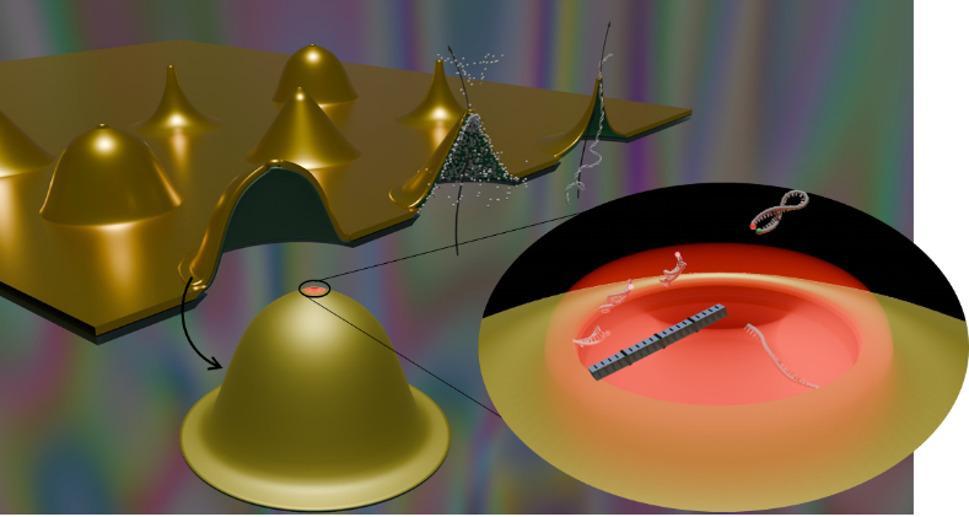

**Supplementary Information:**

The online version contains supplementary material available at 10.1186/s12951-026-04509-9.

## Introduction

Solid-state nanopores have emerged as versatile platforms [1] for single-molecule sensors [2, 3]. Their robustness, chemical stability, and compatibility with semiconductor processing have enabled several applications ranging from DNA sequencing [4, 5] to iontronics [6, 7] and even data-storage readout [8, 9]. Following our earlier work on three-dimensional conical dielectric nanopores, which established a robust platform for electrical and iontronic functionalities [10], here we extend this concept toward three-dimensional plasmonic nanopores designed for optical techniques at the single-molecule level. When metals such as gold are introduced, plasmonic antennas concentrate light into nanoscale hot spots [11], enhancing excitation and emission rates [12] and enabling optical control of single-molecule dynamics [13], gating [14], and guiding chemical reactions at the nanoscale [15]. These plasmonic nanopores therefore provide a unique platform in which electromagnetic, electrical, and thermal fields can be tuned precisely within tens of nanometers [16, 17]. However, direct single-molecule optical interrogation inside metallic nanopores remains challenging because fluorophores operating near metal surfaces experience strong non-radiative decay and fluorescence quenching [18].

Understanding and exploiting this near-field regime requires a method capable of reporting local optical conditions with nanometer precision, ideally one that can reveal both spatial and temporal information on emission processes and kinetics. DNA-PAINT (Points Accumulation for Imaging in Nanoscale Topography) is an ideal candidate technique [19]. An open question is whether stochastic single-molecule techniques such as DNA-PAINT can operate reliably inside confined plasmonic nanopores, where strong near-field enhancement and quenching coexist over only a few nanometers. Based on the transient hybridization between short fluorescent “imager” strands and complementary “docking” sequences immobilized on a surface, DNA-PAINT produces stochastic fluorescence bursts that can be localized with sub-diffraction precision [20, 21]. Its modular chemistry and single-molecule sensitivity make it particularly suitable for probing complex nanostructured interfaces [22], including plasmonic architectures where localized fields and surface chemistry together determine optical behavior [23].

**Fig. 1:**
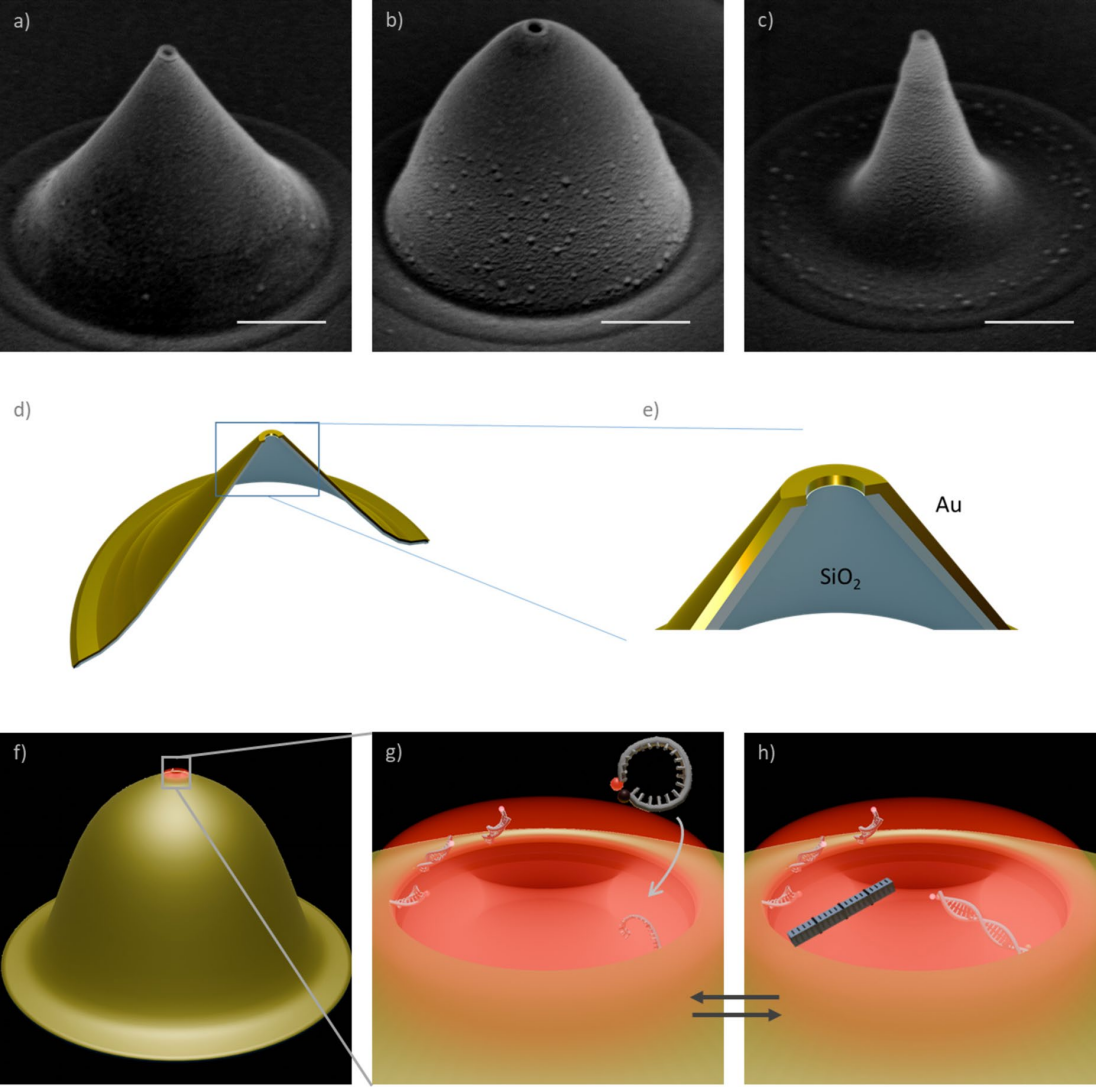
Plasmonic nanopores and optical probing strategies. (top) SEM images of representative conical gold nanopores fabricated on thin membranes, showing reproducible tip morphology and nanoscale apertures at the center. Scale bars: 500 nm. (middle) Schematics of a cross section of the Au nanopore, with detail of the tip of the structure. (bottom) Schematic illustration of the two experimental configurations used in this work: DNA-PAINT imaging, where transient fluorescent events report local optical fields at the nanopore tips, and distance-dependent fluorescence measurements using DNA oligonucleotides of nominal lengths (3 nm, 6 nm, 9 nm) acting as molecular nanorulers. Together, these approaches enable controlled optical interrogation of plasmonic enhancement and quenching within the confined near-field region

In this work, we introduce 3D plasmonic nanopores as an optically active extension of our previously developed dielectric nanopore platform. First, we demonstrate, to our knowledge, the first implementation of DNA-PAINT inside a nanopore, establishing that stochastic single-molecule fluorescence readout can be achieved within a confined metallic nanofluidic environment. Second, we use DNA-based nanorulers to position fluorophores at certain nominal distances from the gold surface, and probe how fluorescence is shaped by the balance between electromagnetic enhancement and metal-induced quenching [24]. We observe a clear distance-dependent response, with an optimal regime at intermediate spacing where enhancement outweighs non-radiative decay, in agreement with the expected behavior of emitters near plasmonic gold nanostructures [25].

Third, we introduce dual-material Au/Si nanopores as a hybrid extension of the plasmonic nanopore platform. Static fluorescence measurements with the 6 nm spacer confirm that these structures remain optically addressable, while Rhodamine 6G measurements reveal a response distinct from that of fully metallic Au nanopores. Simulations further suggest that the hybrid geometry supports an asymmetric electromagnetic field distribution. In addition to modifying the local optical environment [26], such multi-material structures could enable distinct surface chemistries on the metallic and semiconducting regions, opening a route toward multifunctional opto-nanofluidic nanopores.

Figure 1 illustrates the plasmonic nanopore architecture and the combined experimental approaches used in this work. Together, these experiments address whether plasmonic nanopores can support stochastic single-molecule optical interrogation and whether hybrid nanopore architectures can extend this platform toward multimaterial opto-nanofluidic systems.

## Results and discussion

**Fig. 2:**
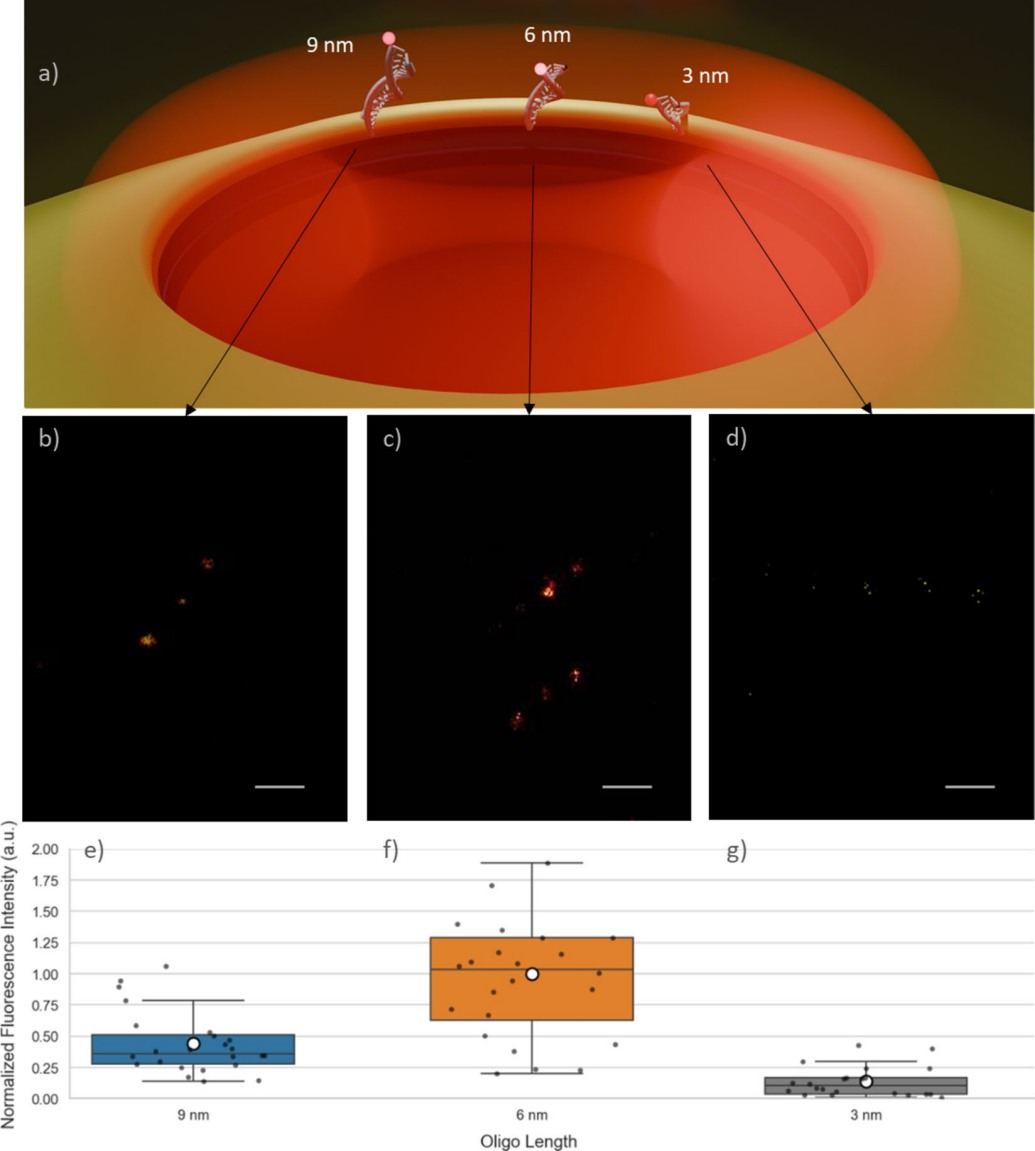
Fluorescence from plasmonic nanopores functionalized with DNA oligos of different lengths with Atto647N on the free end. (**a**) Schematic representation of a plasmonic nanopore showing fluorophores located at distinct distances from the metallic surface, corresponding to different near-field enhancement regions (red shading). (**b-d**) Confocal fluorescence maps of nanopores decorated with ~ 9 nm, 6 nm, and 3 nm oligos, respectively. Bright spots correspond to individual pores with distinct emission levels depending on the DNA oligo length. (**e-g**) Boxplots of the measured fluorescence intensities for each oligo length. Black dots represent individual nanopores and white circles indicate mean values. The non-monotonic intensity dependence confirms a balance between enhancement and quenching mechanisms, defining an optimal intermediate fluorophore-metal spacing for maximum brightness. Scale bars: 2 μm

We first investigated how the fluorophore-metal separation distance influences emission strength in plasmonic nanopores by functionalizing identical pores with DNA oligonucleotides of three different lengths (as detailed in Table S1, S2 and S3), therefore positioning the dyes at controlled separations from the metallic surface (Fig. 2a) [27]. Each sample was prepared by the same surface chemistry and labeling protocol to ensure that only the spacer length varied. The nanopores used for the optical measurements had nominal aperture diameters of approximately 40 ± 5 nm based on SEM estimates (Figure S1). Cross-sectional views are shown in Figure S2. Ionic-current measurements performed on a representative Au pore (Figure S3) yielded a conductance value consistent with a nanopore aperture in the same nominal size range as estimated from SEM. Because this estimate is obtained using a flat-pore model with an effective pore length that does not directly correspond to the actual conical geometry, it is used only as a qualitative consistency check rather than as a direct geometrical determination [28].

Using the three DNA spacers of different length, we observed a clear dependence of fluorescence intensity on the nominal fluorophore-metal separation, as shown in Fig. 2b-g (additional examples shown in Figure S6). The shortest linker (3 nm) produced weak emission, consistent with optical quenching by the metal; whereas the intermediate 6 nm spacer yielded the highest fluorescence intensity, representing the regime where electromagnetic enhancement appears to outweigh metal-induced quenching. The longest 9 nm linker resulted in a slightly lower intensity than the 6 nm case, consistent with near-field decay with increasing separation [29]. Here, 3/6/9 nm denote nominal spacer lengths rather than exact dye–metal distances, and the effective separation may differ due to linker geometry, strand flexibility, conformational tilting, and local surface roughness [30]. The non-monotonic trend is interpreted as arising from competing effects. At the shortest nominal separation the fluorophore experiences the strongest dipole-metal coupling but also the strongest metal-induced losses, leading to weak emission. At the intermediate nominal separation, the fluorophore still samples the enhanced field while being less affected by short-range losses, resulting in the highest fluorescence; at the largest nominal separation, both the local field intensity and the dipole-metal coupling are reduced, and the fluorescence decreases again. This interpretation is qualitatively supported by dipole-power calculations in Supporting Information Sect.  4, where the total decay-rate modification decreases from 105 to 47 to 27 for the nominal 3, 6, and 9 nm positions, respectively.

Despite the three pore shapes producing distinct ionic behaviors [10], their fluorescence readouts at 640 nm were broadly comparable under our experimental conditions. Electromagnetic simulations indicate that the convex geometry may provide somewhat lower electromagnetic enhancement and could therefore appear less bright. However, a much larger experimental dataset would be required to distinguish shape-dependent effects with confidence, especially given the substantial variability observed even across measurements with the three spacer lengths.

Normalizing the fluorescence to the mean 6 nm intensity highlights the relative efficiency of each spacer length: 3 nm yields ~ 0.15 ×⟨I₆ₙₘ⟩, 6 nm is set to 1, and 9 nm gives ~ 0.5 ×⟨I₆ₙₘ⟩. This non-monotonic response is consistent with the enhancement/quenching interplay near plasmonic antennas: 3 nm is expected to be strongly quenched, 6 nm maximizes enhancement, and 9 nm experiences weaker electromagnetic enhancement due to field decay in line with typical distance-dependent plasmon–emitter interactions [25, 31].

Variations in total intensity could also be influenced by differences in the number of fluorophores associated with each nanopore. In addition to pure optical effects, mass transport and packing during functionalization can bias total intensity. Shorter oligos have smaller hydrodynamic radii and higher diffusion coefficients, which facilitate reaching the surface over a fixed incubation time, and the adsorption/association rate scales with the diffusion coefficient so faster diffusers deliver more strands to the pore within the same time [32]. Their smaller steric footprint can also permit denser packing in the confined rim region. Together, these factors could increase the number of emitters per pore even if per-emitter brightness is reduced. Measurements capable of resolving the number of fluorophores per pore, such as single-molecule counting, or probing photophysics independently of intensity, such as lifetime measurements [33, 34], would help clarify whether the observed trends arise purely from optical distance effects or from variations in labeling density. Importantly, the 3 nm spacer remains the dimmest case despite these transport and packing factors potentially favoring higher loading of shorter oligonucleotides, which supports the interpretation that metal-induced quenching dominates at short fluorophore-metal separation. Although the number of measurements per spacer condition is limited and the variability between nanopores is non-negligible, the overall trend is consistent across the dataset, with the 6 nm spacer giving the highest fluorescence, the 3 nm spacer the lowest, and the 9 nm spacer an intermediate response.

Overall, this distance-dependent response provides useful design guidelines for plasmonic nanopore optimization. Avoiding the quenching zone directly adjacent to the metal, targeting intermediate fluorophore-metal separations where electromagnetic enhancement outweighs quenching, and accounting for potential variations in labeling density are key for maximizing fluorescence yield. We therefore use simulations to map the spatial distribution of |E|² and identify regions of stronger local field enhancement within the nanopore geometry.

**Fig. 3:**
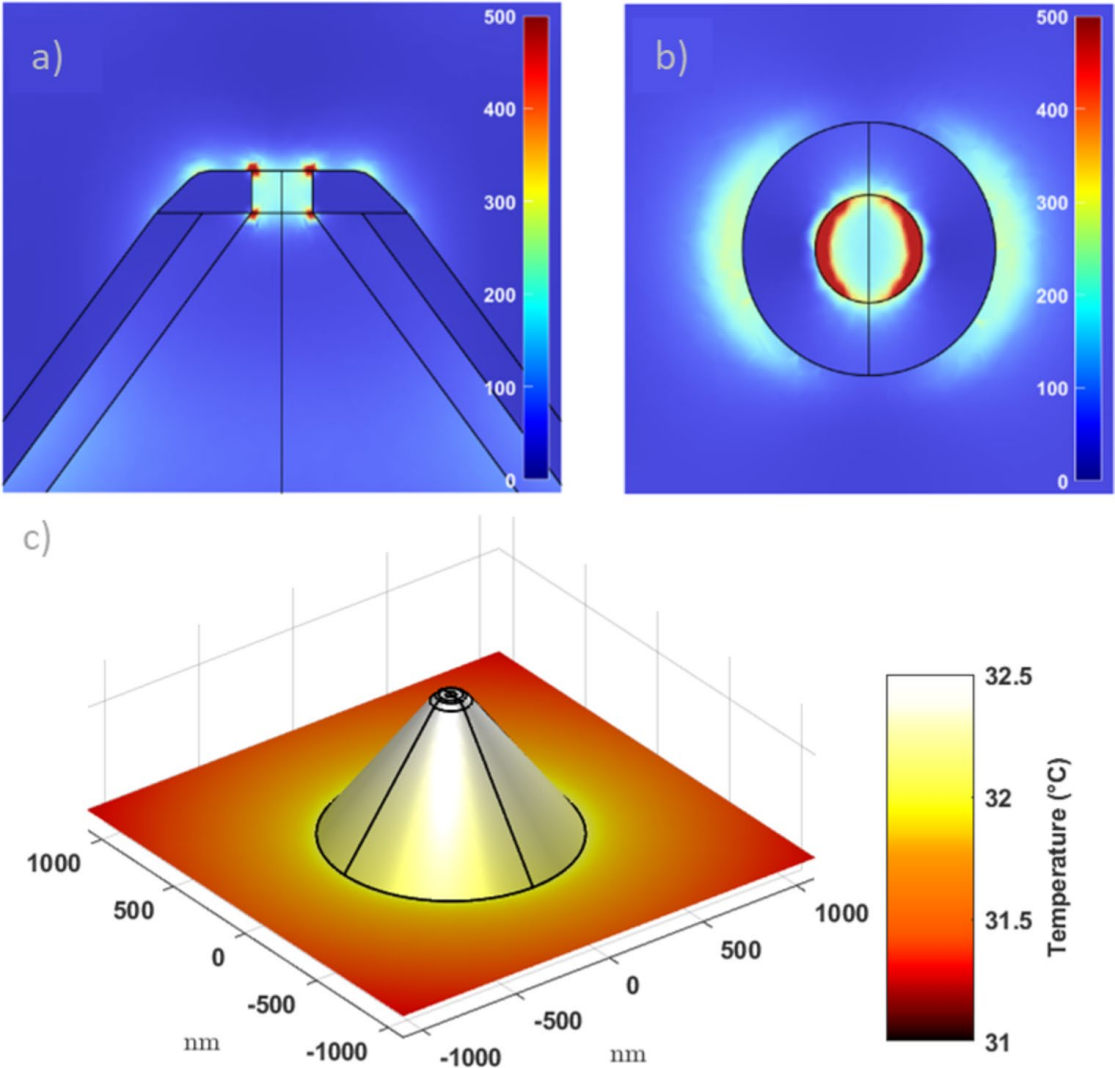
Simulated electric field enhancement (∣∣^2^/∣_0_∣^2^) at λ = 634 nm for 40 nm 3D nanopores. (**a**,** b**) Cross-sectional and top-view maps of the field intensity for a gold-coated conical nanopore. The simulations were performed at λ = 634 nm, a wavelength selected close to the experimental illumination at 640 nm. The mode produces strong, symmetric confinement around the pore, with maximum intensity within a few nanometers from the metal surface. (**c**). Steady-state temperature map at λ = 634 nm, irradiance = 15 µW µm⁻², bulk T₀ = 20 °C; color scale shows T

The simulations show appreciable electromagnetic field enhancement at wavelengths close to the red excitation used experimentally. As shown in Fig. 3a, b the field is strongly enhanced within the first few nanometers from the metal surface, and then the field rapidly decays with distance. This spatial trend, together with the stronger short-distance total decay-rate modification obtained from the dipole-power calculations (Supporting Information Sect.  4), is qualitatively consistent with the experimentally observed higher fluorescence at intermediate nominal separations, where emitters can still sample the enhanced field while reducing the impact of metal-induced losses [32]. In contrast to the electrical characteristics previously reported for similar 3D conical nanopores [10], the simulated near-field distribution was only moderately affected by the wall angle of the conical structure, although the convex geometry showed somewhat lower enhancement than the straight and concave cases. The simulations shown here were performed under linearly polarized excitation. The distance-dependent fluorescence measurements were acquired by standard confocal microscopy without intentional polarization control, whereas the DNA-PAINT measurements were performed under circularly polarized excitation. Additional simulations under right- and left-handed circular polarization, included in the Supporting Information (Figure S18), show the same qualitative field-confinement pattern and hotspot localization as the linearly polarized case. The simulations are therefore used as qualitative guidance for the electromagnetic field distribution rather than as exact reproductions of the experimental excitation conditions. Additional dipole-power calculations (Supporting Information Sect.  4) show that the simulated total decay-rate modification decreases from 105 to 47 to 27 for the nominal 3, 6, and 9 nm positions, respectively, indicating weaker dipole–metal coupling with increasing distance. Because this quantity reflects the total decay-rate change rather than fluorescence output alone, it is used here only as qualitative support for stronger metal-induced losses at short distance.

Plasmonic metals irradiated near resonance do not only lead to a strong increase in the local electromagnetic field, but also produce significant thermal losses. A fraction of the absorbed energy is converted into heat, and therefore a noticeable increase in temperature near the metal nanostructures occurs as shown in Fig. 3c in a 3D perspective rendering; a corresponding cross-section is shown in Figure S12. Additional simulations including rounded pore edges show that finite curvature reduces the absolute enhancement substantially relative to the ideal sharp-edge model (Figure S17). The corresponding temperature distributions are shown in Figures S14-S16 although the qualitative spatial distribution of the field remains similar. These results indicate that the idealized model overestimates the field magnitude near the metal boundary, while still capturing the main features of the confinement pattern.

In the nanopore geometry the thermal contribution can be relevant, as the area coated by the plasmonic metal (gold) extends beyond the nanopore, and fully encompasses the hot spot region at the pore aperture. Figure 3c shows that our calculations yield a maximum local temperature of approximately 32.5 °C around the nanopore under irradiation at 634 nm and 15 µW/µm^2^, from a bulk temperature of 20 °C. Such local heating may influence experiments involving biomolecules and DNA, particularly in relation to hybridization and photophysical behavior. Local heating is not expected to substantially disrupt the Au-thiol anchoring of the fluorophore-bearing strand over the duration of the experiment, since this strand is thiol-tethered to the Au surface. Its main effect would instead be to influence the stability and rigidity of the spacer duplex, thereby broadening the distribution of effective fluorophore-metal distances, particularly for the shortest duplexes.

**Fig. 4:**
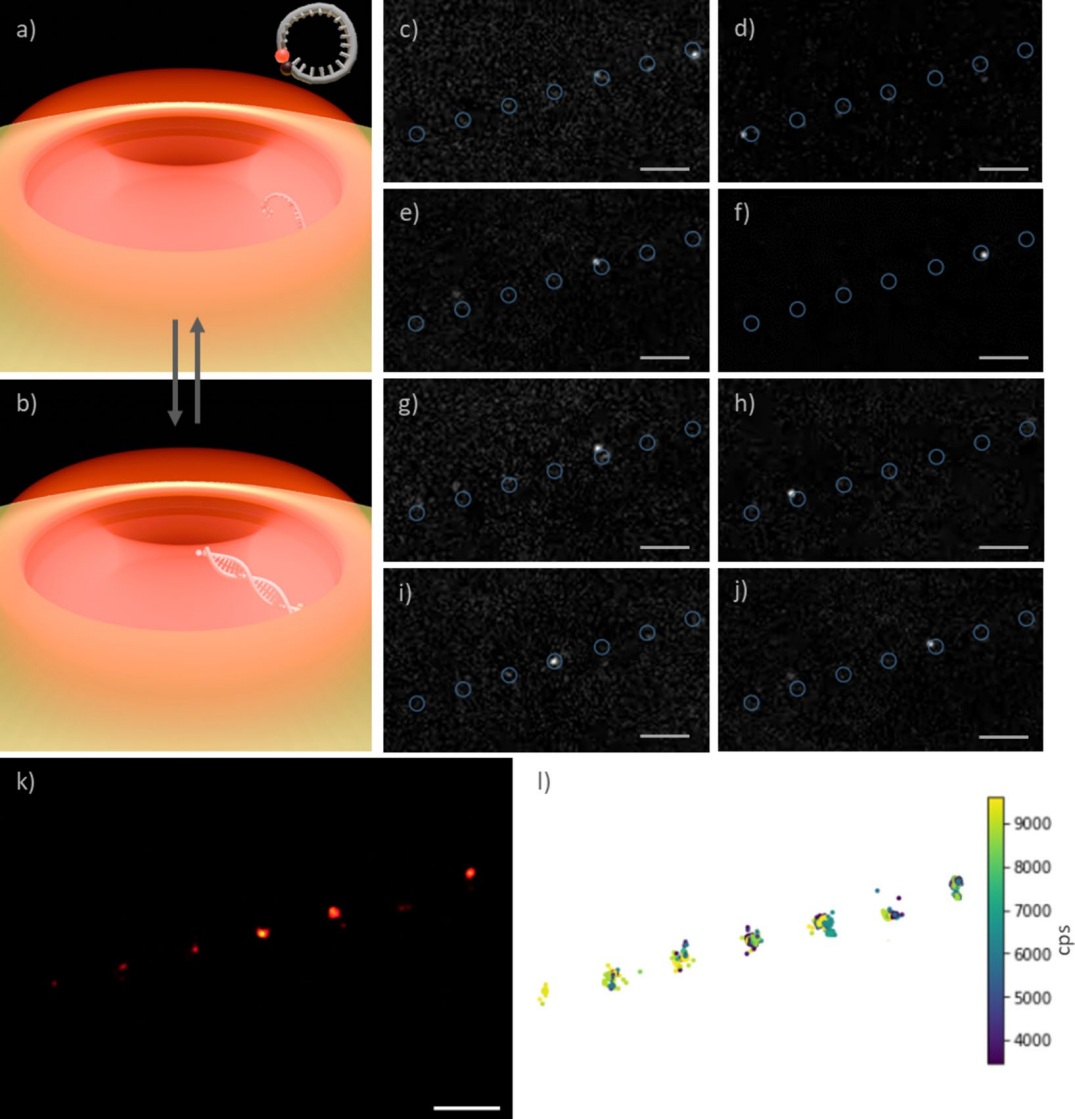
DNA-PAINT on plasmonic nanopores. (**a**,** b**) Schematic representation of the DNA-PAINT process occurring inside a plasmonic nanopore. Transient hybridization between fluorescently labeled imager strands and complementary docking sequences at the pore tip generates stochastic fluorescence bursts. (**c-j**) Snapshots from wide-field fluorescence imaging, showing individual binding events occurring on an array of nanopores (highlighted by blue circles). (**k**) Localization map reconstructed with Picasso [20], displaying the positions of single-molecule events accumulated over several minutes. (**l**) Emission rate (counts per second) per localization, revealing the distribution of emission intensities across nanopores. The enhanced brightness in selected pores indicates local electromagnetic field amplification in or near the nanopore. Scale bars: 2 μm

DNA-PAINT imaging was then used to investigate whether transient single-molecule fluorescence events could be resolved in plasmonic nanopores functionalized with DNA docking strands. Upon introducing complementary fluorescent imager strands in solution, transient hybridization events produce characteristic fluorescence bursts at the nanopore openings (Fig. 4a, b). Unlike conventional DNA-PAINT experiments performed at the glass–water interface under total internal reflection fluorescence (TIRF), here the active nanostructures are located several hundred nanometers above the coverslip, within the metallic membrane, as shown in Figure S8. Consequently, evanescent illumination cannot selectively excite bound imagers (as in TIRF), and measurements were carried out under wide-field epi-illumination conditions. To suppress fluorescence background from freely diffusing strands, we employed fluorogenic imager oligonucleotides carrying a dye–quencher pair that remain dark in solution and become fluorescent only upon hybridization to the docking sequence at the nanopore tip (as detailed in Table S4). This fluorogenic design ensures that detectable fluorescence arises only from hybridized imager strands, whereas unbound strands in solution remain effectively dark under the imaging conditions used here [35]. The temporal nature of these events enables single-molecule localization and reconstruction of the nanopore-associated emission pattern. At our chosen oligo length (15 bp imager) and attachment geometry, the fluorophore is expected to reside at a distance of ~ 5–7 nm from the metal surface [30], within a region of strong near-field enhancement.

The observed DNA-PAINT dynamics are shown in Video S1, and representative frames in Fig. 4c-j show discrete, diffraction-limited spots corresponding to single binding events occurring on individual nanopores. The consistent alignment of these events with the predefined array confirms that hybridization occurs preferentially at the nanopore positions. Each pore acts as a localized reaction site, where the brightness of single binding events is influenced by the local optical environment, while the event frequency is governed by DNA-PAINT kinetics set by imager concentration and sequence. Minor variations between pores arise from differences in local accessibility, diffusion, or local optical conditions.

Localization analysis using Picasso [20] (Fig. 4k) revealed dense clusters of events at the nanopore positions. The reconstructed map confirms that DNA-PAINT signals originate predominantly from the pores, with minimal background elsewhere on the substrate. Notably, despite the overnight incubation applied to the entire surface, we did not observe meaningful PAINT signal on the surrounding flat substrate when focusing the optics on the plane of the substrate (Figure S9). This supports the effectiveness of our localized functionalization protocol [12] for probing optical effects at the nanopore apex.

To quantify the emission characteristics, we first examined the integrated photon count per event, i.e. the total number of photons detected during each transient hybridization (Figure S10a). The integrated signal depends on both brightness and event duration. The imager-docking pair used here was designed to yield characteristic binding durations of about one second, consistent with standard DNA-PAINT kinetics [35, 36]. In our data, dwell times ranged from ~ 0.1 s to > 10 s, corresponding to total photon yields between 10³ and 5 × 10⁵ photons per event. The correlation between photon count and dwell time confirms that long-lived events, even if moderately bright, can accumulate photon totals even larger than short, intense bursts (Figure S10c) [37].

From these quantities, we derived the emission rate (counts per second) for each event to visualize spatial variations across the nanopore array (Fig. 4l). Each cluster corresponds to a single nanopore and displays characteristic intensity levels, typically between 4 × 10³ and 9 × 10³ photons s⁻¹ (5–95% range). The resulting map highlights heterogeneity in emission rate and brightness, consistent with local differences in excitation field strength, molecular orientation, or nanoscale geometry at the pore tips. The fitted background showed no correlation with event intensity (*r* < 0.1; see Figure S11), indicating that brightness variations are not due to local background and are instead consistent with differences in the local optical response of the plasmonic structures [20, 38]. 

Enhanced local heating in high-field regions could, in principle, accelerate unbinding, leading to shorter dwell times but higher brightness [39]. However, dwell times represent true binding kinetics only if the dye remains emissive throughout the bound state. Under strong excitation, photobleaching can prematurely terminate fluorescence, particularly near plasmonic hot spots where local field is strongest. As a result, apparent unbinding events may partly reflect bleaching rather than dissociation. Distinguishing these effects will require extended analyses of possible thermoplasmonic influences [40, 41] as described for similar plasmonic nanopore systems [12]. The corresponding dwell-time distributions are reported in Figure S10b, but further studies with controlled excitation and larger datasets will be necessary to disentangle these factors.

These results demonstrate that DNA-PAINT can be implemented in plasmonic nanopores and can serve as a sensitive reporter of the local nanoenvironment within confined plasmonic structures [42]. To our knowledge, this represents the first experimental realization of DNA-PAINT within solid-state nanopores, establishing a new platform that combines single-molecule localization microscopy with nanofluidic confinement. Future experiments combining photon-count statistics, dwell-time distributions, and temperature mapping will help to clarify the relative contributions of optical enhancement, local heating, and kinetic effects in shaping the observed fluorescence dynamics.

**Fig. 5:**
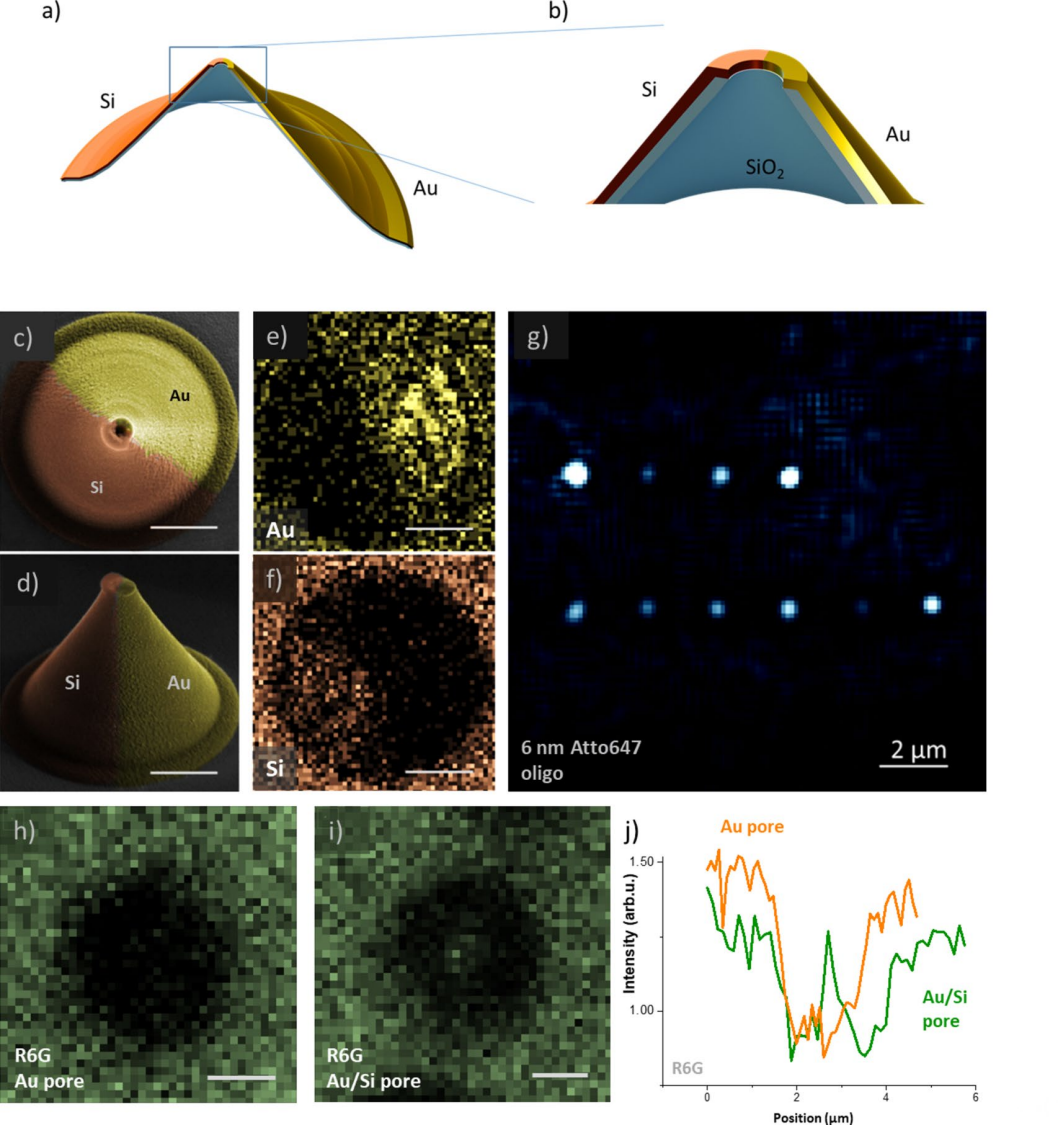
Structural and optical characterization of dual-material Au/Si nanopores. (**a**,** b**) Cross-sectional schematics of the hybrid conical nanopore geometry, showing the laterally combined Au and Si regions. (**c**,** d**) Representative false-colored SEM images of a hybrid nanopore in top and tilted view, where silicon is shown in orange and gold in yellow to highlight the two materials. (**e**,** f**) Representative false-colored EDX elemental maps, confirming lateral segregation of Au (yellow) and Si (orange) within the structure. (**g**) Representative fluorescence image of hybrid nanopores functionalized with the 6 nm Atto647N oligo. (**h**,** i**) Confocal Rhodamine 6G fluorescence images for an Au pore and an Au/Si pore, respectively. (**j**) Corresponding intensity line profiles showing a distinct fluorescence response for the two pore geometries (green: Au/Si pore, orange: Au pore). Scale bars: 500 nm in (**c-f**), 1 μm in (**h, i**)

Finally, we explored whether introducing a second material could extend the plasmonic nanopore platform toward hybrid architectures with modified optical response [43]. For this purpose, we fabricated dual-material conical nanopores in which gold and silicon are laterally combined within the same structure, bringing a plasmonic material and a high-index semiconductor into a single confined geometry (Fig. 5) [44, 45]. The cross-sectional schematics in Fig. 5 illustrate the asymmetric conical geometry of the Au/Si nanopore, while the corresponding SEM and EDX images are false-colored to distinguish the two materials, with silicon shown in orange and gold in yellow, which further confirm the lateral segregation between gold and silicon, validating the hybrid design. The SEM images show well-defined Au/Si interfaces and reproducible pore geometries ranging from shallow cones to sharper tips (Fig. 5c, d, Figure S4), and a cross section view is shown in Figure S5a. Again, ionic-current measurements on a representative Au/Si pore (Figure S5b) gave a conductance value consistent with a nanopore aperture in the same nominal size range. As above, this estimate is interpreted only as a qualitative consistency check, since the conductance analysis relies on a flat-pore approximation with an effective pore length rather than on the true conical geometry [28]. Upon functionalization with the 6 nm Atto647-labeled DNA spacer, confocal fluorescence imaging reveals localized emission from the hybrid nanopore array (Fig. 5g), confirming that these dual-material structures remain optically active after functionalization [46].

To determine whether the dual-material architecture modifies the optical response, we also compared Rhodamine 6G fluorescence in a fully metallic Au pore and in an Au/Si hybrid pore using a 532 nm confocal setup. Representative confocal images and corresponding line profiles (Fig. 5h–j) show a distinct fluorescence response for the two geometries: for the gold only architecture, the pore remains dark, while the Au/Si dual nanopore shows an increase in intensity near the center of the structure (additional examples shown in Figure S7). At present, this difference cannot be attributed uniquely to a single mechanism. It may reflect differences in local electromagnetic enhancement and quenching between the Au and Au/Si geometries, but contributions from different adsorption or interaction behavior of the dye on the silicon region cannot be excluded. In particular, fluorophores associated with the silicon side may experience a different local environment and reduced quenching compared with those interacting only with gold.

Electromagnetic simulations of the dual-material nanopore, currently available for 634 nm excitation (Figure S13), indicate an asymmetric field distribution, with the strongest enhancement localized on the metallic side, while the silicon region contributes differently to the overall optical response at that wavelength [47]. Although these simulations are not wavelength-matched to the 532 nm Rhodamine 6G experiment, they support the view that the hybrid geometry introduces an intrinsically asymmetric optical environment. This configuration therefore provides an additional design parameter for shaping the local optical response and may, in the future, support multimaterial opto-nanofluidic operation by combining the distinct optical and surface properties of the two materials.

These dual-material nanopores introduce a versatile route toward multi-material nanopore architectures, combining plasmonic field enhancement with the distinct optical and surface-chemical properties of semiconductors. Their asymmetric composition provides an additional design parameter for tuning nanoscale optical response and may open opportunities for future multifunctional sensing and opto-nanofluidic applications [48].

## Conclusions

We have demonstrated, to our knowledge for the first time, the implementation of DNA-PAINT within plasmonic nanopores, introducing a single-molecule optical readout directly coupled to a nanofluidic system. The transient binding of fluorescent imagers at the nanopore positions supported the effectiveness of the localized functionalization protocol and demonstrated that localized fluorescence activity can be resolved within the confined plasmonic geometry. This work complements the previously explored electronic functionalities of three-dimensional conical nanopores by adding an optical pathway to probe localized electromagnetic field enhancement at the nanoscale.

By employing DNA spacers of controlled length, we further investigated how nominal fluorophore-metal separation regulates the balance between electromagnetic enhancement and quenching. The observed emission trend (minimal brightness at 3 nm, a maximum at 6 nm, and reduced intensity at 9 nm) provides a practical design guideline for optimizing optical response in metallic nanopores. These results highlight that emitters placed within an intermediate distance regime can benefit most strongly from local field enhancement, while those closer to the surface are more strongly affected by non-radiative losses.

Finally, the introduction of dual-material Au/Si nanopores expands the platform toward multifunctional architectures with modified optical response. By combining plasmonic and semiconducting regions within a single conical geometry, these structures provide an additional design parameter for tailoring local field distributions and potentially enabling distinct surface chemistries on the two regions. The observed fluorescence differences between Au and Au/Si pores, together with the asymmetric field distributions predicted by simulations, support the relevance of this hybrid geometry for future opto-nanofluidic architectures.

Collectively, our results establish plasmonic nanopores as a promising nanoscale platform for correlating optical fields, thermal effects, and molecular kinetics in confined geometries. The integration of DNA-based probes provides a strategy to engineer and interrogate nanostructured interfaces with molecular precision, opening new directions for single-molecule sensing, plasmon-enhanced spectroscopy, and nanopore-based optical readout schemes. Future work will correlate fluorescence lifetime, local temperature, and ionic measurements to better resolve the coupled optical, thermal, and nanofluidic effects present in hybrid nanopores.

### Experimental methods

#### Substrate and nanopore fabrication

Three-dimensional oxide nanopores were fabricated on SiN/Si substrates by combining spin coating of photoresist, focused ion beam (FIB) drilling, atomic layer deposition (ALD) of dielectric material (SiO₂), and a final annealing step [10]. A 20 nm Au layer was then deposited to form a plasmonic nanoantenna at the pore tip, with a 3–5 nm Cr or Ti adhesion layer between the dielectric and metal. Dual-material nanopores were obtained by directional metal deposition: Au was evaporated at 90° to coat one side of the conical pores, followed by Si deposition after rotating the substrate 180°, yielding laterally asymmetric dielectric-plasmonic structures.

#### Surface chemistry and tip-selective functionalization

Before immobilization on the Au-coated nanopores, thiolated DNA oligonucleotides were reduced with tris(2-carboxyethyl)phosphine hydrochloride (TCEP) to generate free thiol groups for Au–thiol binding. Briefly, the thiolated strands were incubated with freshly prepared TCEP following the standard reduction protocol at room temperature. After reduction, excess TCEP was removed using Amicon centrifugal filters, and the purified oligonucleotides were used immediately for nanopore functionalization.

Functionalization followed an established nanopore decoration protocol [12] adapted to DNA oligonucleotides in TAE buffer. Chips were plasma-activated for 5 min from the back (dielectric) side, 1x TAE buffer (pH 8) was added to the top side, and a DNA-linker solution was applied on the back side (1 µM in 1x TAE buffer). Docking strands or oligo nanorulers were incubated briefly (0.5 min) for distance-series labeling. After incubation, samples were rinsed in TAE buffer. Under these asymmetric conditions, oligonucleotide attachment localized predominantly at the nanopore tip with negligible adsorption on the surrounding flat substrate.

#### DNA-PAINT

Docking strands were incubated overnight, and rinsed thereafter, in the same buffer conditions as the distance series oligos. Imaging strands were introduced during acquisition under standard PAINT conditions at a concentration of 10 nM. Tip and base regions were imaged separately by shifting the focal plane 1–2 μm. Short-incubation tests (minutes) were performed for comparison with nanoruler experiments.

Imager: 5′-/Atto633N/AAGTTGTAATGAAGA/BHQ₂/-3′.

Docking: 5′-/ThiolMC6-D/TTATCTCCTATACAACTTCC/-3′.

#### Oligo nanorulers

Three double-stranded DNA constructs positioned the fluorophore at around 3, 6, and 9 nm from the metal through defined poly-A/T spacers. After short incubations (30 s–1 min), confocal fluorescence maps were recorded around individual pores.

3 nm: 5′-/ThiolMC6-D/AAAAAAAAAA/Atto647N/-3′ + 5′-/TTTTTTTTTT/-3′.

6 nm: 5′-/ThiolMC6-D/AAAAAAAAAAAAAAA/Atto647N/-3′ + 5′-/TTTTTTTTTTTTTTT/-3′.

9 nm: 5′-/ThiolMC6-D/AAAAAAAAAAAAAAAAAAAA/Atto647N/-3′ + 5′-/TTTTTTTTTTTTTTTTTTTT/-3′.

#### Optical measurements

Fluorescence intensity maps of nanoruler-functionalized nanopores were acquired on a Nikon A1R+ confocal microscope using a 100× oil-immersion objective (NA 1.49) and 640 nm excitation (50 µW). Emission was collected through standard filter sets and recorded under identical conditions for all samples to enable quantitative comparison of fluorescence enhancement versus distance.

Rhodamine 6G fluorescence measurements comparing Au and Au/Si pores were performed in the confocal setup under 532 nm excitation, whereas the electromagnetic simulations shown for the hybrid geometry were carried out at 634 nm. The samples were immersed in a 10 µM Rhodamine 6G solution.

DNA-PAINT measurements were performed on a custom-built wide-field fluorescence microscope based on an inverted Olympus IX83 body. Samples were illuminated in epi-configuration with a 640 nm laser (Laser Quantum) providing a Gaussian beam and circularly polarized excitation. Fluorescence was collected through a 100× oil-immersion objective (NA 1.5, Olympus), filtered by a 532/640 nm dual-band set (Chroma), and detected on a CMOS camera (ORCA-Fusion, Hamamatsu).

#### Numerical simulations

Three-dimensional electromagnetic simulations were performed in COMSOL Multiphysics using realistic conical geometries including the dielectric and Au layers. Near-field intensity distributions were computed at λ = 634 nm, a simulated wavelength selected close to the experimental red excitation, to identify zones of field enhancement.

## Electronic Supplementary Material

Supplementary Material 1

Supplementary Material 2

## Data Availability

The datasets generated and/or analyzed during the current study are available from the corresponding author on reasonable request.
