# Supplementary material for: Three-dimensional plasmonic nanopores for DNA-PAINT and dual-material Au/Si architectures
Source: J Nanobiotechnology. 2026 May 8;24:605. doi: 10.1186/s12951-026-04509-9 (PMC13330494; doi:10.1186/s12951-026-04509-9)
Supplement: Supplementary file 1 — Supplementary Material 1 [file 12951_2026_4509_MOESM1_ESM.docx]

Three-Dimensional Plasmonic Nanopores for DNA-PAINT and Dual-Material Au/Si Architectures

German Lanzavecchia^1,2^, Anastasiia Sapunova^1,3^, Alan M. Szalai^4^, Shukun Weng^1,3^, Ali Douaki^1,2^, Makusu Tsutsui^5^, Roman Krahne^1^, Guillermo Acuna^4*^, Denis Garoli^1,2*^

1. Optoelectronics, Istituto Italiano di Tecnologia, 16163 Genova, Italy;
2. Dipartimento di Scienze e Metodi dell’Ingegneria, Università degli Studi di Modena e Reggio Emilia, 43122 Reggio Emilia, Italy
3. Università degli Studi di Milano-Bicocca, 20126, Milano, Italy
4. Department of Physics, University of Fribourg, Fribourg CH-1700, Switzerland
5. SANKEN, The University of Osaka, 495 Osaka, Ibaraki 567-0047, Japan

Emails: [denis.garoli@unimore.it](mailto:denis.garoli@unimore.it), [Guillermo.acuna@unifr.ch](mailto:Guillermo.acuna@unifr.ch)

# Section 1: DNA oligo details

*Table S1: 3 nm nanoruler oligo (poly-A/T spacer). The top strand is thiolated (Thiol-C6) for Au attachment and the dye (Atto647N) is at the opposite end. The complementary poly-T strand is shown below. This sequence defines the short (~3 nm nominal) spacer used in the distance-series measurements.*

| 5’ | Thiol | A | A | A | A | A | A | A | A | A | A | Atto647N | 3’ |
| --- | --- | --- | --- | --- | --- | --- | --- | --- | --- | --- | --- | --- | --- |
| 3’ | | T | T | T | T | T | T | T | T | T | T | 5’ | |

*Table S2: 6 nm nanoruler oligo (poly-A/T spacer). The top strand is thiolated (Thiol-C6) for Au attachment and the dye (Atto647N) is at the opposite end. The complementary poly-T strand is shown below. This sequence defines the intermediate (~6 nm nominal) spacer used in the distance-series measurements.*

| 5’ | Thiol | A | A | A | A | A | A | A | A | A | A | A | A | A | A | A | Atto647N | 3’ |
| --- | --- | --- | --- | --- | --- | --- | --- | --- | --- | --- | --- | --- | --- | --- | --- | --- | --- | --- |
| 3’ | | T | T | T | T | T | T | T | T | T | T | T | T | T | T | T | 5’ | |

*Table S3: 9 nm nanoruler oligo (poly-A/T spacer). The top strand is thiolated (Thiol-C6) for Au attachment and the dye (Atto647N) is at the opposite end. The complementary poly-T strand is shown below. This sequence defines the long (~9 nm nominal) spacer used in the distance-series measurements.*

| 5’ | Thiol | A | A | A | A | A | A | A | A | A | A | A | A | A | A | A | A | A | A | A | A | Atto647N | 3’ |
| --- | --- | --- | --- | --- | --- | --- | --- | --- | --- | --- | --- | --- | --- | --- | --- | --- | --- | --- | --- | --- | --- | --- | --- |
| 3’ | | T | T | T | T | T | T | T | T | T | T | T | T | T | T | T | T | T | T | T | T | 5’ | |

*Table S4: Imager and docking strands used for DNA-PAINT. The imager carries Atto633N at the 5′ end and BHQ2 at the 3′ end; the docking strand is Thiol-C6 modified for Au attachment. Intentional mismatches (to enforce transient binding) are indicated as underlined in the table.*

| 5’ | Atto633N | | **A** | **A** | **G** | **T** | **T** | **G** | **T** | **A** | ***A*** | ***T*** | **G** | ***A*** | **A** | **G** | **A** | BHQ2 | 3’ | | | |
| --- | --- | --- | --- | --- | --- | --- | --- | --- | --- | --- | --- | --- | --- | --- | --- | --- | --- | --- | --- | --- | --- | --- |
| 3’ | C | C | **T** | **T** | **C** | **A** | **A** | **C** | **A** | **T** | ***A*** | ***T*** | **C** | ***C*** | **T** | **C** | **T** | A | T | T | Thiol | 5’ |

# Section 2: 3D plasmonic nanopores and static fluorescence


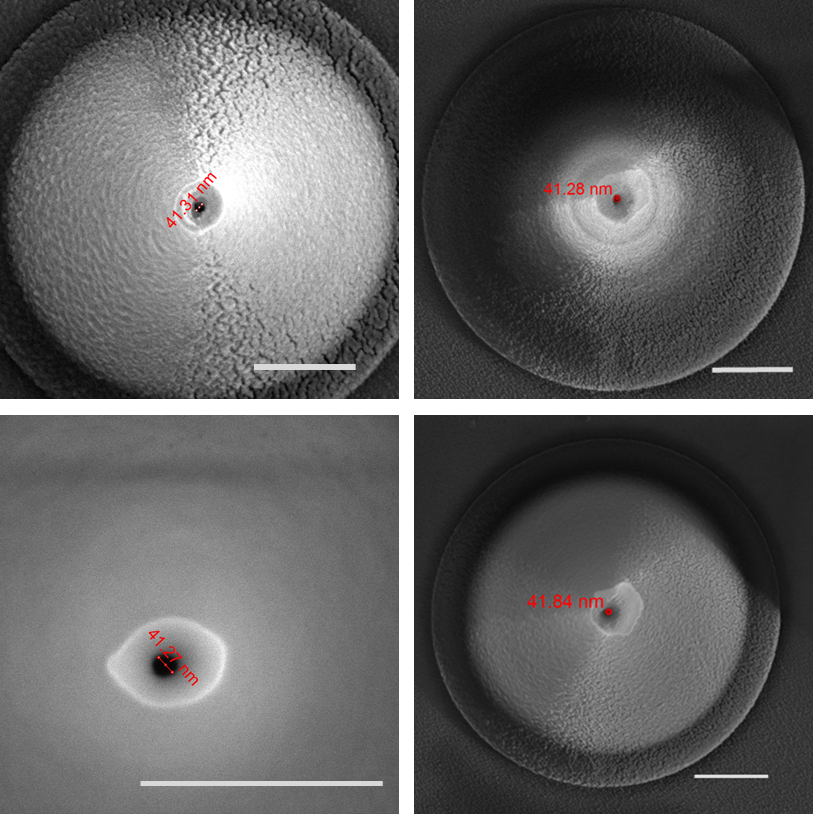


Figure S1. Representative nanopore diameters measured in SEM


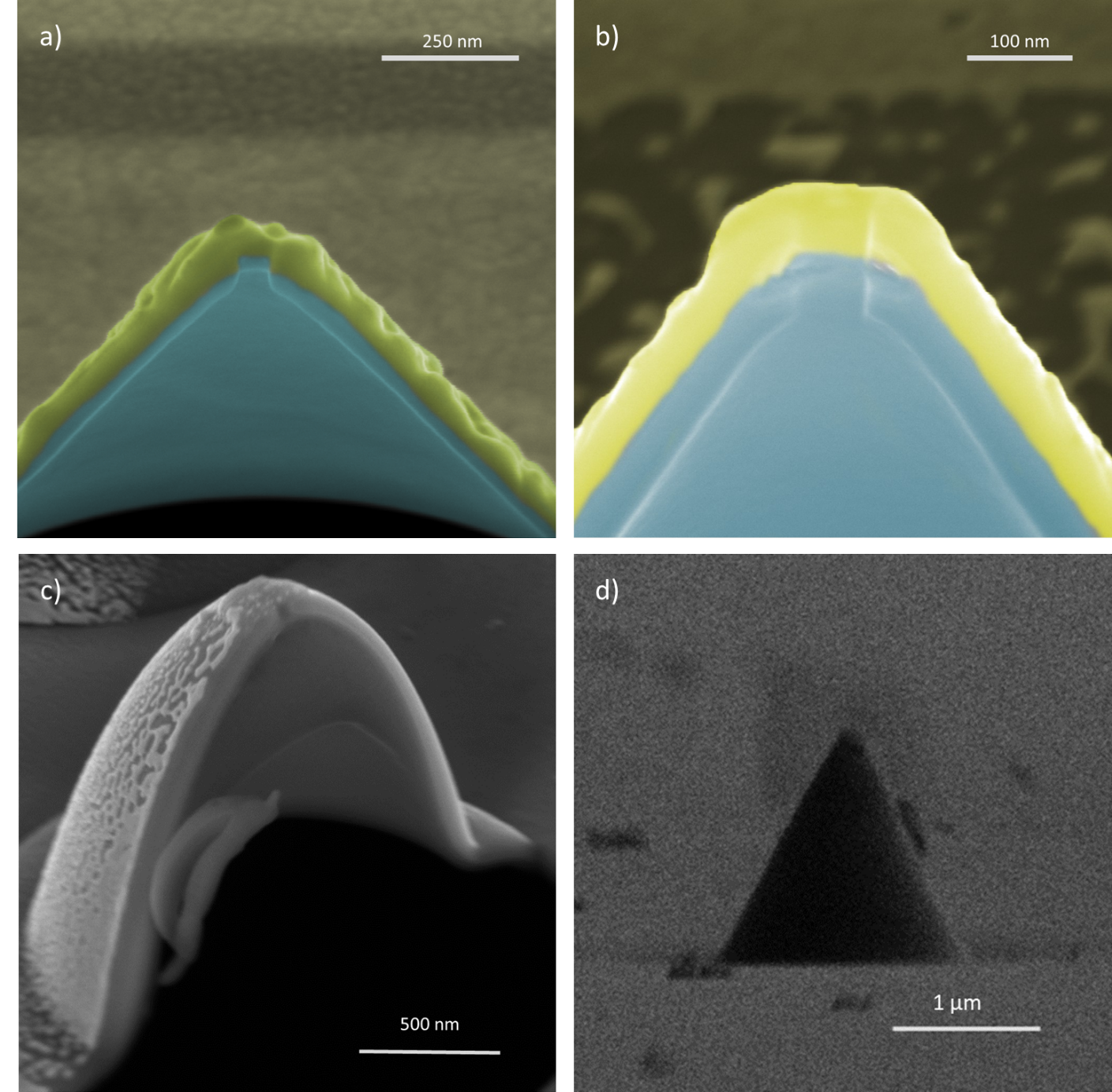


Figure S2. a-c) Cross-section of different nanopores showcasing the layers a,b) false-colored in yellow for gold, and blue for silicon oxide. d) Side view of an Au nanopore at 90°

Figure S3. Representative ionic-current measurement of an Au nanopore in 10 mM KCl. The conductance-based estimate is consistent with a nanopore aperture in the same size range as the nominal SEM diameter, although it is derived using an effective flat-pore model and is therefore used only as a qualitative consistency check. Calculated diameter was 47 ± 3 nm.


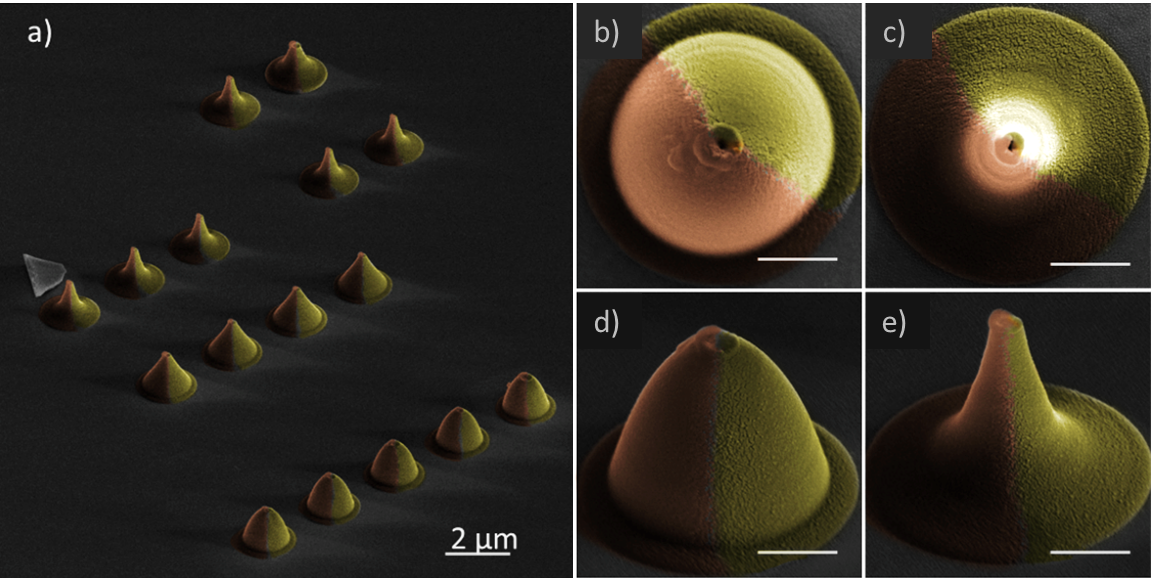


Figure S4. Additional SEM images of the different geometries prepared with Au/Si dual architecture, showcasing in yellow the gold area and in orange the silicon region.


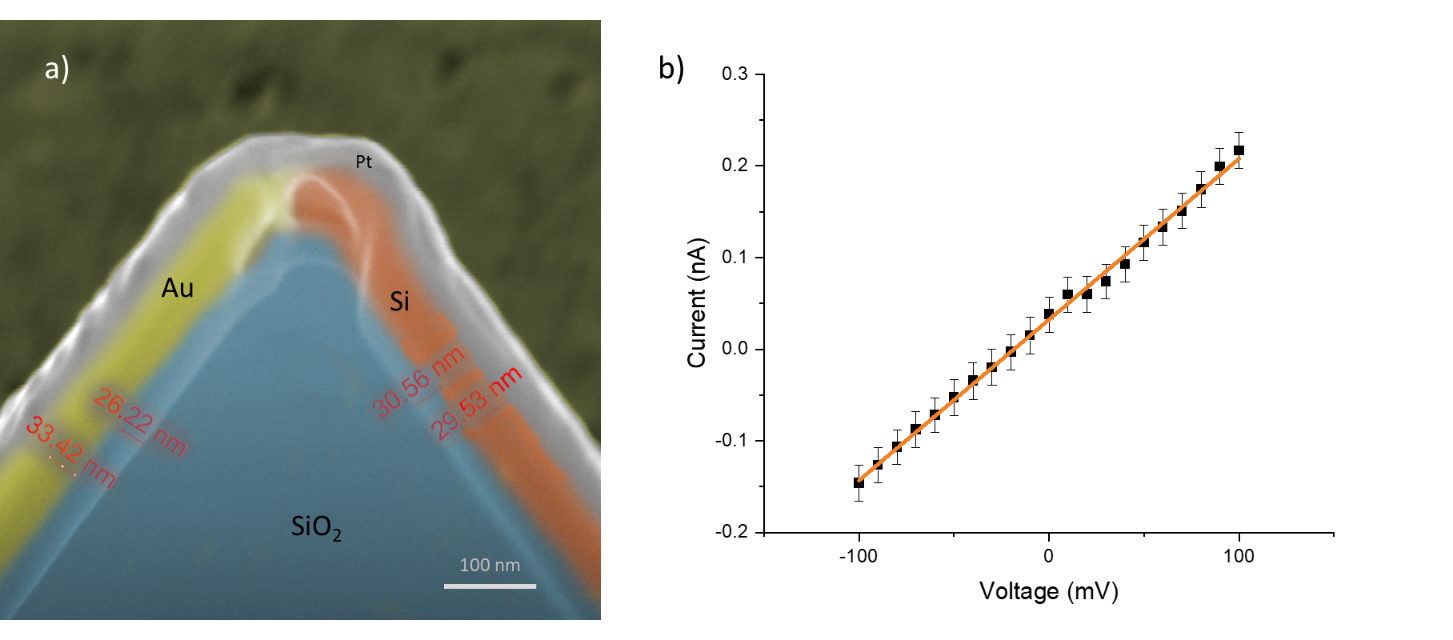


Figure S5. a) Cross-section SEM image of Au/Si nanopore false-colored: Au in yellow, Si in orange and SiO_2_ in blue. b) Representative ionic-current measurement of an Au/Si nanopore. The conductance-based estimate is consistent with a nanopore aperture in the same size range as the nominal SEM diameter, but is used only as a qualitative consistency check because the model assumes an effective flat-pore geometry. The calculated diameter was 36 ± 2.5 nm.


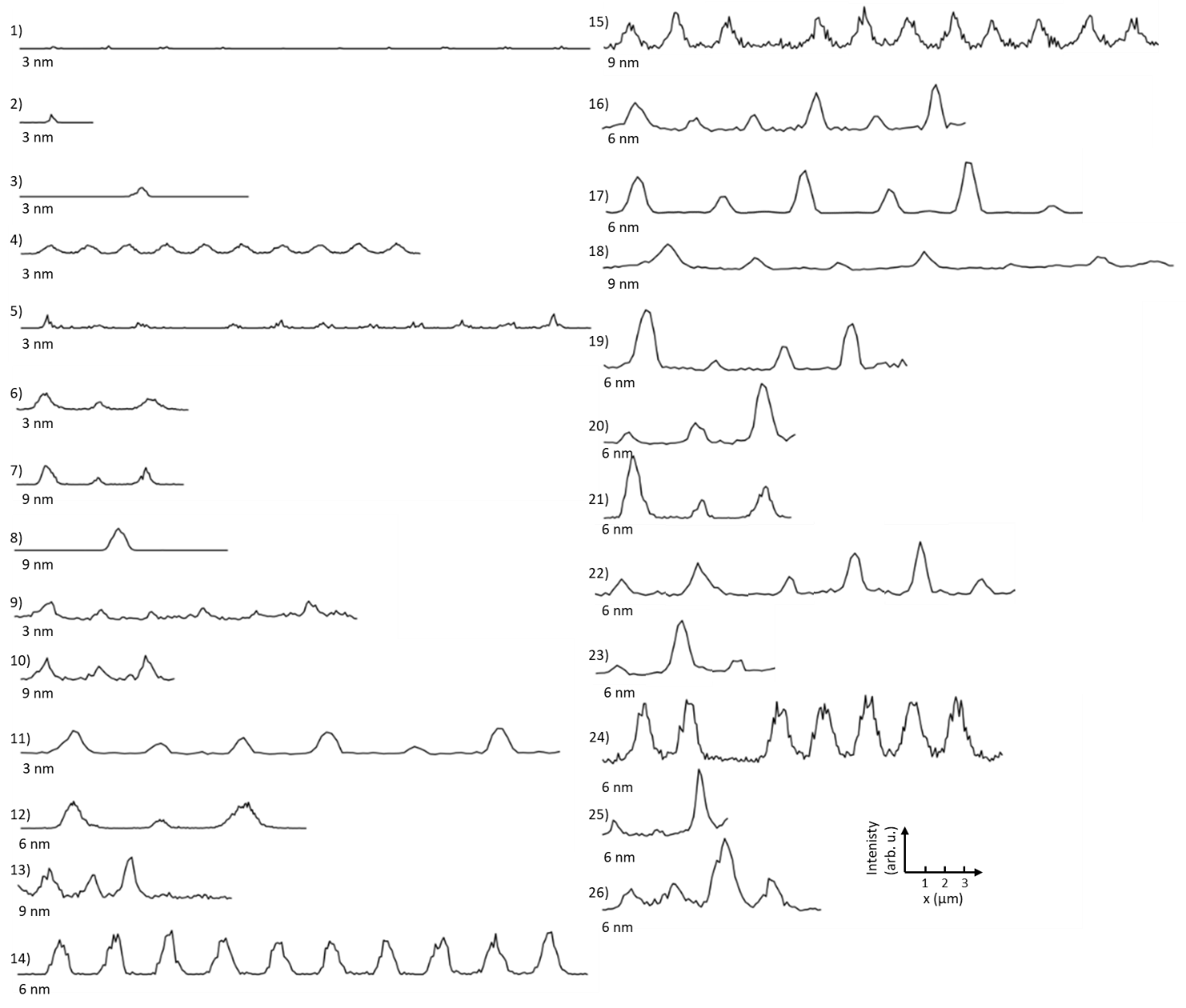


Figure S6. Intensity (arbitrary units) profiles of different samples with aligned nanopores decorated with the three different length oligos with Atto647N. The x-axis corresponds to the lateral position across the fluorescence profile and is expressed in micrometers (µm). In series 1,3,4,5,14,15,18,24, the first peak corresponds to a convex nanopore and the second to a concave nanopore. In all other positions the nanopores were straight.

*
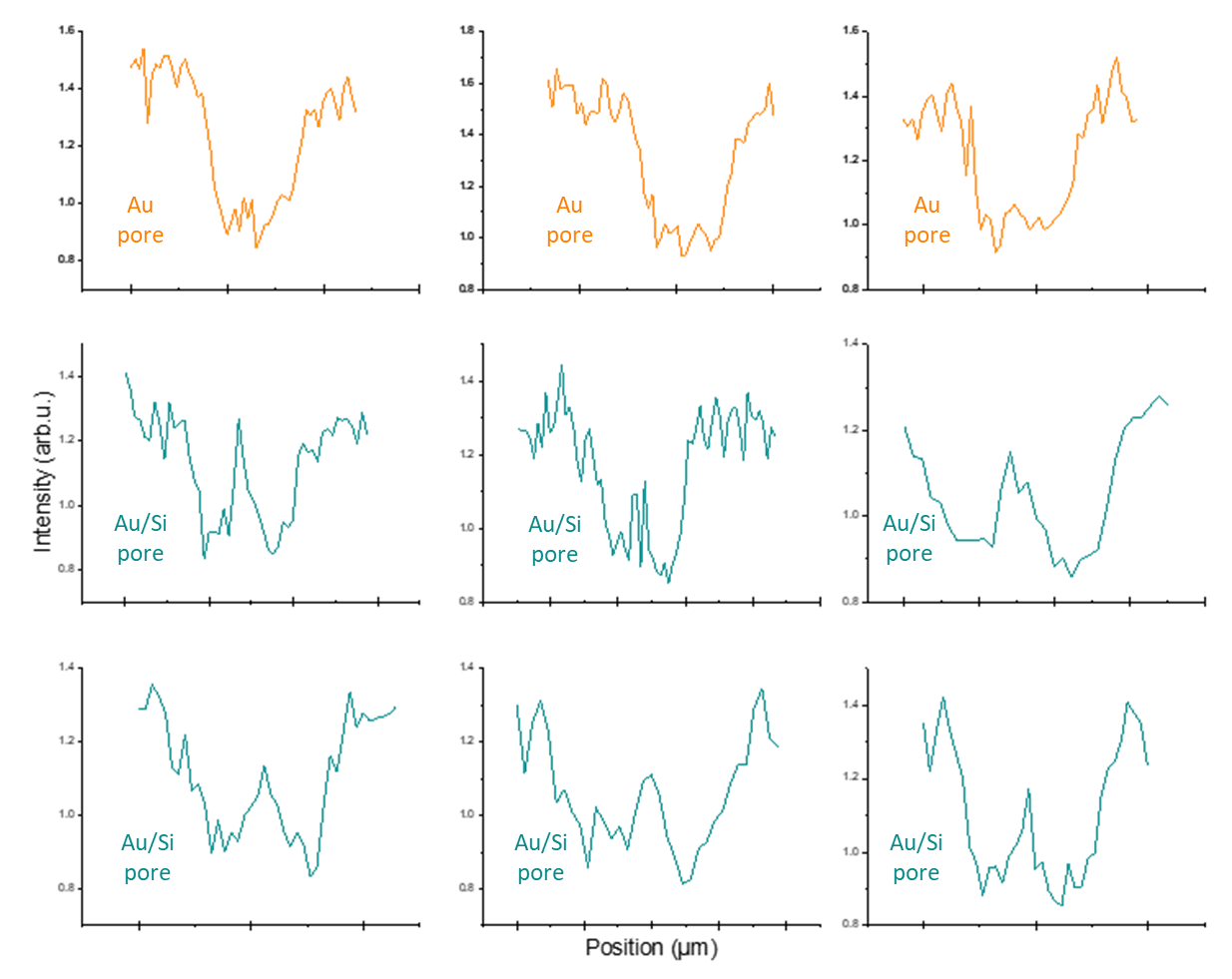
*

Figure S7. Additional line profiles of Rhodamine 6G fluorescence in Au and Au/Si nanopores. Representative confocal fluorescence intensity line profiles acquired under 532 nm excitation for fully metallic Au pores (top row) and dual-material Au/Si nanopores (middle and bottom rows). Consistent with the examples shown in Figure 5h–j, the Au and Au/Si geometries exhibit different fluorescence responses, with the Au/Si pores frequently showing a less suppressed or locally enhanced central signal. These examples support the conclusion that the dual-material architecture modifies the local optical response.

# Section 3: DNA-PAINT

#
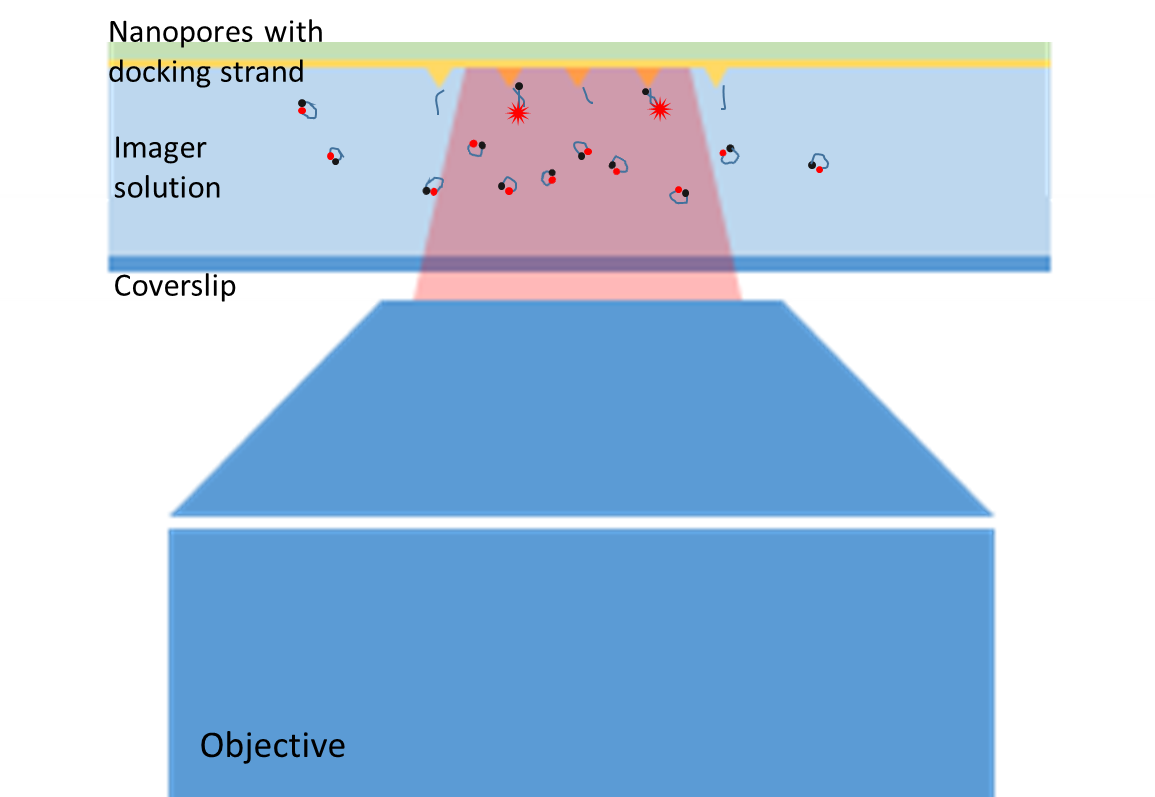


Figure S8. Schematics of the setup used for the DNA-PAINT measurement illustrating the need for a dye-quencher pair. The dye–quencher design ensures that detectable fluorescence originates predominantly from hybridized imager strands, while freely diffusing strands remain effectively dark under the imaging conditions used.


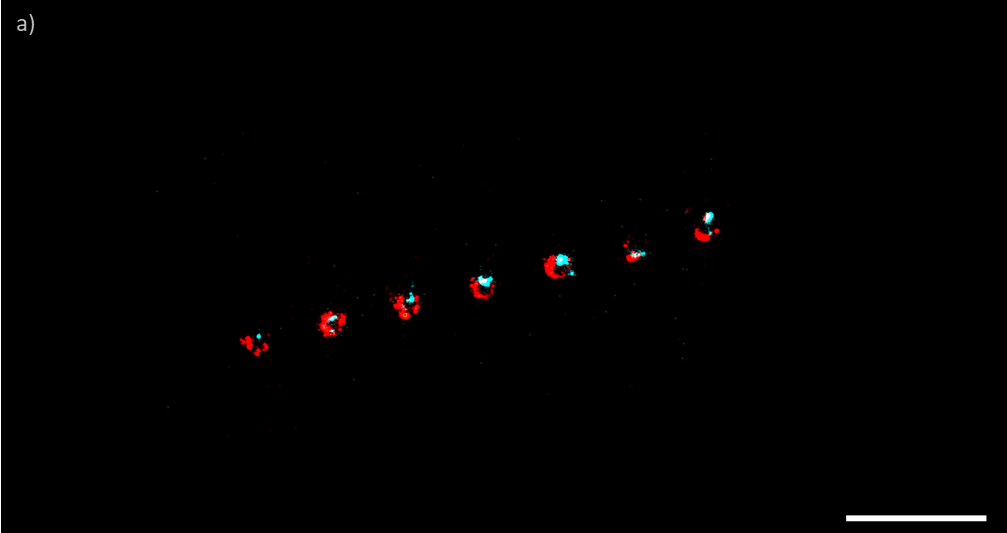


*Figure S9.* DNA-PAINT localizations reconstructed with Picasso, acquired both at the pore-tip focal plane (cyan) and at the base/substrate plane (red), showing negligible decoration of the surrounding flat substrate during functionalization.


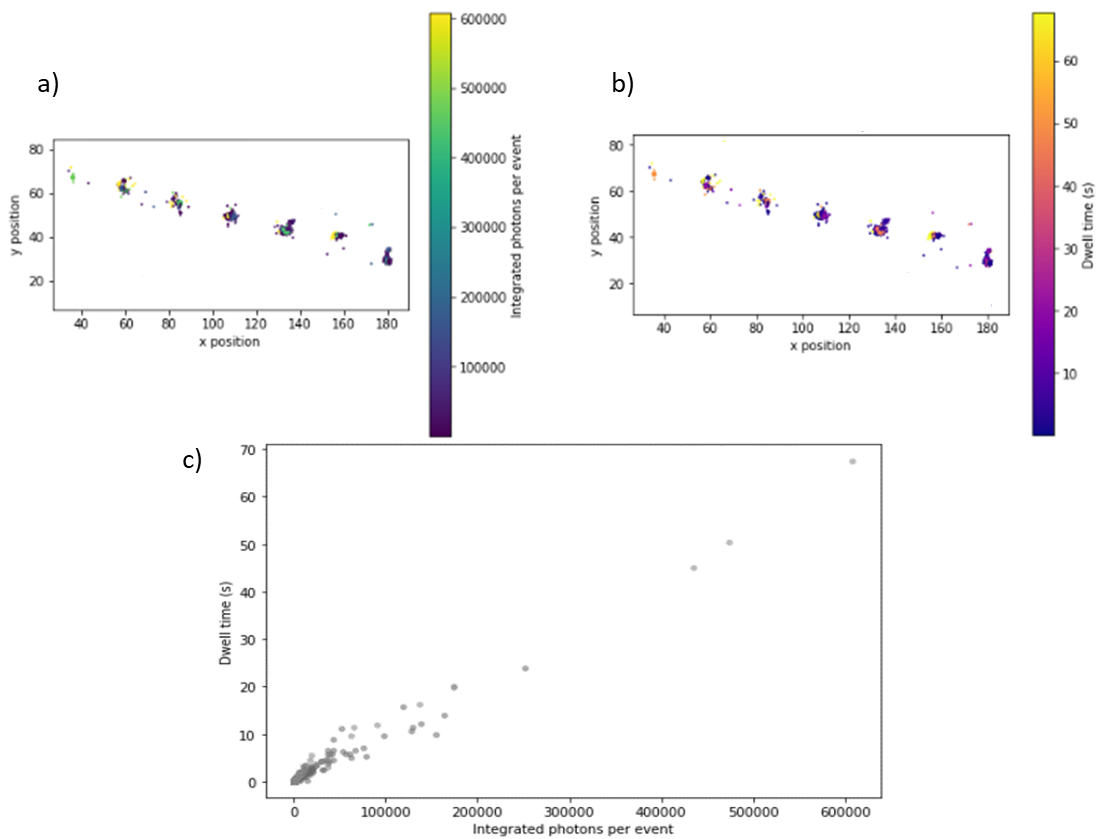


Figure S10. Event map colored by a) integrated photon count and b) dwell time. c) shows the variation of dwell time vs integrated photon count per event.


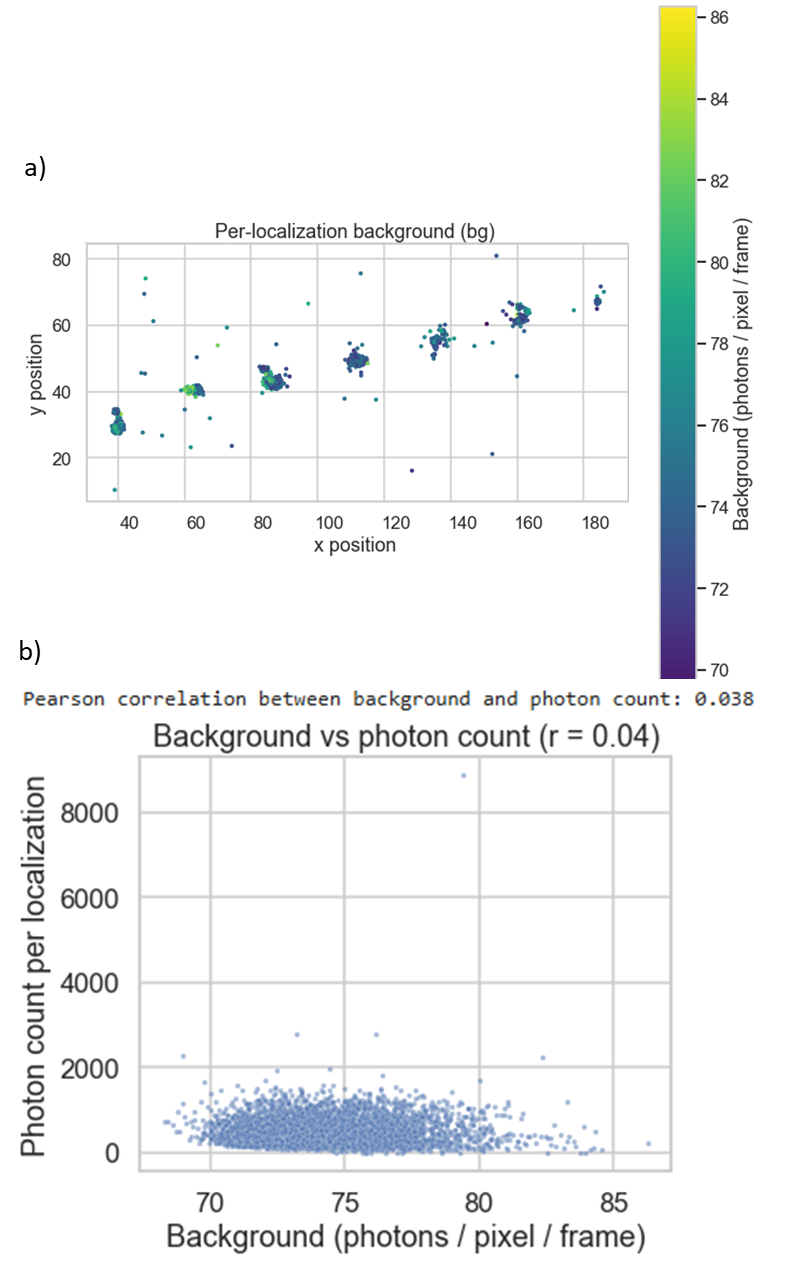


Figure S11. a) Map showing the background per event calculated in Picasso,^1^ and b) the correlation between the background and the photon count per event.

# Section 4: Simulations


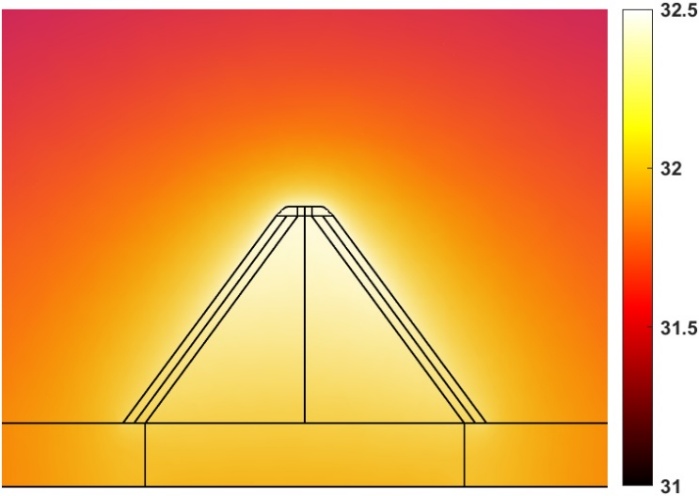


Figure S12. Cross-sectional maps of the simulated temperature distribution at λ = 634 nm, irradiance = 15 µW µm⁻² (≈1.5×10⁷ W m⁻²), bulk T₀ = 20 °C; color scale shows T.


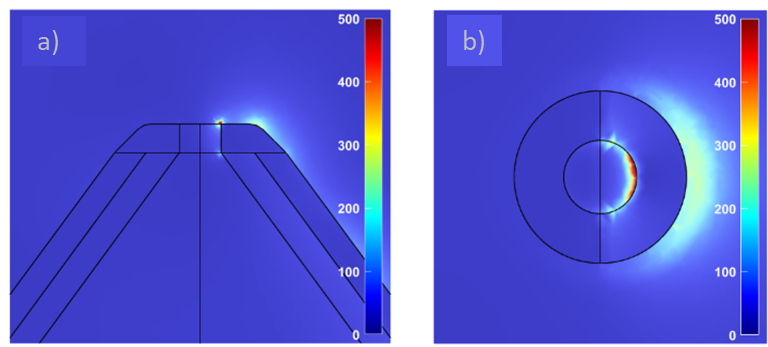


Figure S13. Simulated electric field enhancement (∣𝐸∣^2^/∣𝐸_0_∣^2^) at λ = 634 nm for dual-material nanopores. (a, b) Cross-sectional and top-view maps of the field intensity for a dual-material nanopore combining gold and silicon. The near field is asymmetrically localized toward the metallic side, while the dielectric region shows minimal enhancement at this wavelength.

Electromagnetic field enhancement, temperature distribution and dipole decay-rate modification were calculated in a frequency domain using COMSOL Multiphysics software. The nanopores were illuminated by a plane electromagnetic wave that propagated in the x direction perpendicular to the surface of the nanopore.

All nanopores are characterized with a diameter of 40 nm and located on a silicon nitride substrate with a thickness of 200 nm. The height of all pores, based on SEM images, was around 1400 nm.

The height and width of the simulated box, surrounded by a 1000 nm thick PML layer, are 10000 and 6000 nm, respectively.

The excitation of the electromagnetic field was set via the Port condition in the Electromagnetic Waves, Frequency Domain (ewfd). The obtained distribution of electromagnetic loss density in the plasmonic material was used as a heat source in Heat Transfer in Solids and Fluids (ht). Two physics were coupled through the multiphysics interface of the Electromagnetic Heating.

To correctly calculate the enhancement of the electromagnetic field inside the nanopore and the temperature distribution, a tetrahedral mesh was used. The smallest elements were used inside the nanopore and near its walls (1–10 nm), as well as in the vicinity of the dipole source (0.1–0.5 nm). In the remaining simulation domain, larger mesh elements were applied to reduce computational costs.

To compare more directly with the DNA-PAINT excitation conditions, additional simulations were performed under left- and right-handed circularly polarized illumination. Circular polarization was implemented at the excitation port as two orthogonal electric-field components with a phase shift of π/2, (E = (E₀, ±iE₀, 0)). For both the Au and Au/Si nanopores, the resulting electromagnetic field distributions and intensity-enhancement maps showed the same qualitative field distribution and hotspot localization for the two handednesses. This behavior is consistent with the non-chiral geometry of the plasmonic nanopores, for which the electromagnetic response is not expected to depend on the handedness of circular polarization.

**Calculation of dipole power dissipation and decay-rate change**

To model the emission of a fluorescent source, an electric dipole was placed inside the nanopore at nominal distances of 3, 6, and 9 nm from the nanopore wall. The total power dissipated by the dipole was calculated by integrating the Poynting vector over the surface of a small sphere (radius 1 nm) centered on the dipole position:

S = ∮ S · dA

where S is the Poynting vector and the integration is performed over the closed surface surrounding the dipole.

The total dipole power calculated in the presence of the nanopore was compared with that of the same dipole in a homogeneous medium without the metal nanostructure. The ratio of these two quantities gives the change in the total decay rate of the dipole near the nanostructure:

Γ / Γ₀ = S / S₀

where S is the total dipole power in the presence of the nanopore and S₀ is the total dipole power of the same source in a homogeneous environment. Calculations were performed for three mutually orthogonal dipole orientations, with the dipole moment aligned along the x, y, and z axes. The final value of the decay-rate modification was obtained by averaging the results for the three dipole orientations.

The simulated total decay-rate modification decreases from 105 at the nominal 3 nm position to 47 at 6 nm and 27 at 9 nm, indicating progressively weaker dipole–metal coupling with increasing distance from the nanopore wall. Because this quantity represents the total decay-rate change, it should not be interpreted directly as fluorescence enhancement or quenching efficiency. Nevertheless, the much larger value at short distance is consistent with stronger metal-induced losses near the metal surface and qualitatively supports the experimentally observed suppression of fluorescence for the shortest spacer.


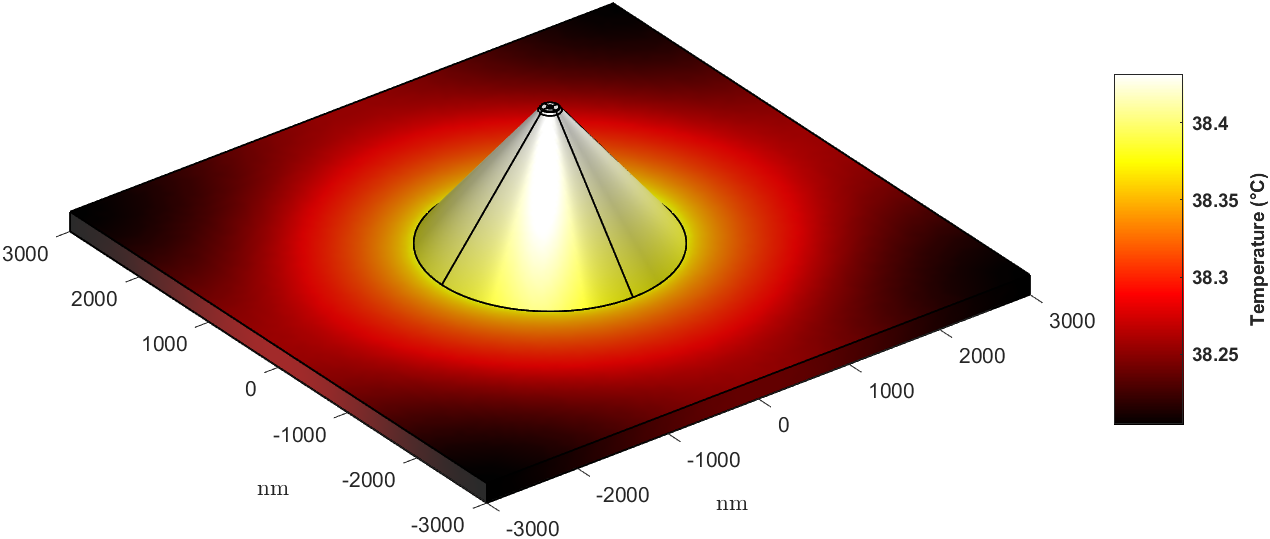


Figure  S14. Simulated temperature distribution at λ = 634 nm for plasmonic nanopores (straight)


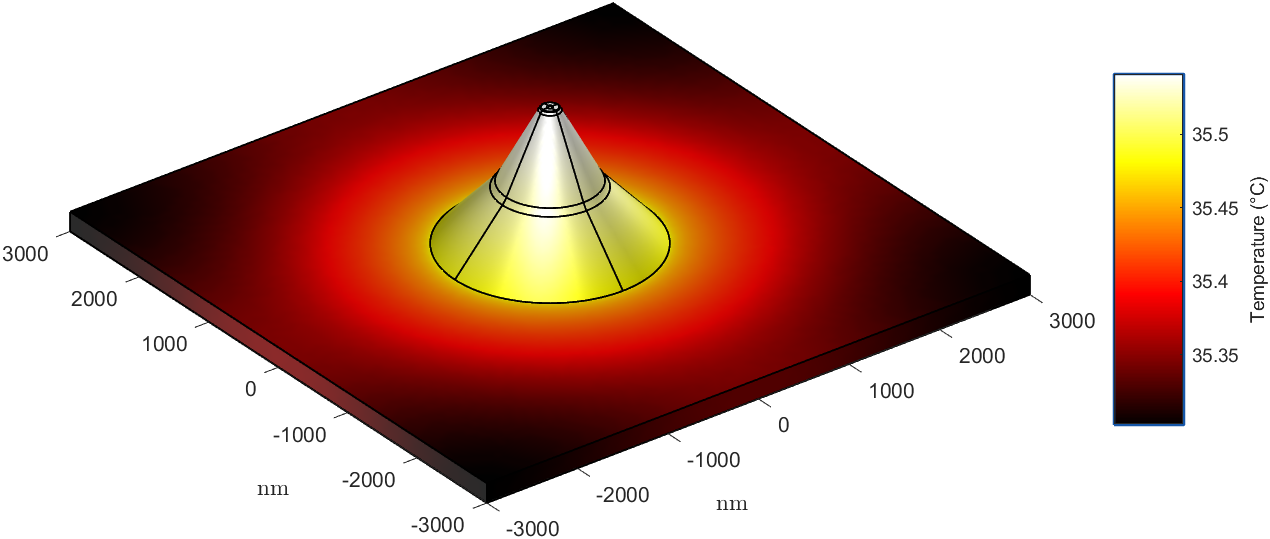


Figure  S15. Simulated temperature distribution at λ = 634 nm for plasmonic nanopores (concave)


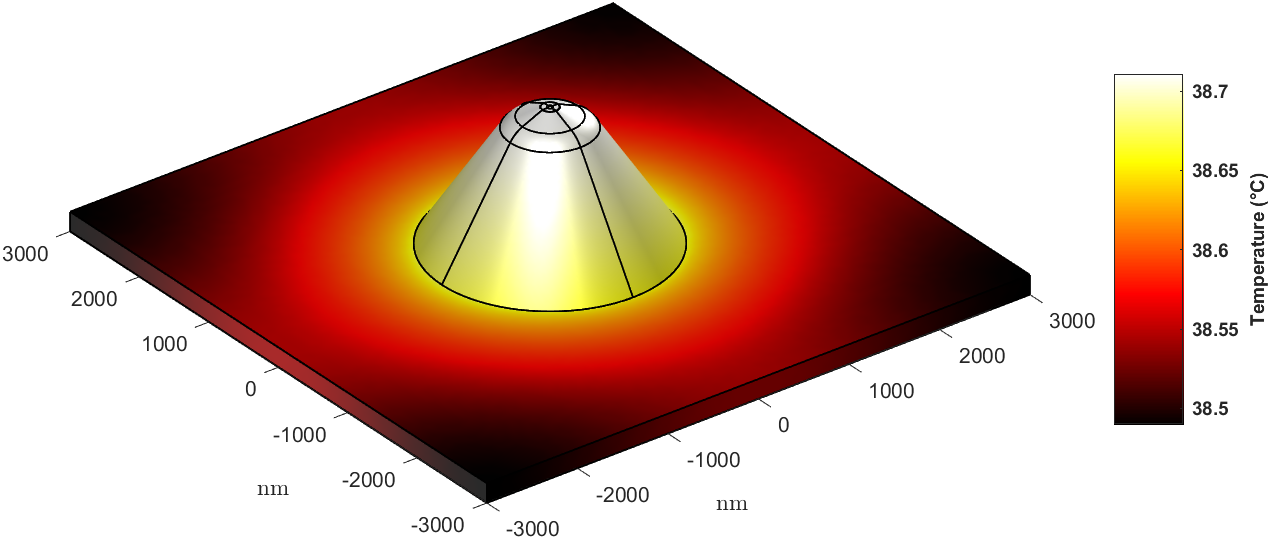


Figure  S16. Simulated temperature distribution at λ = 634 nm for plasmonic nanopores (convex)

| 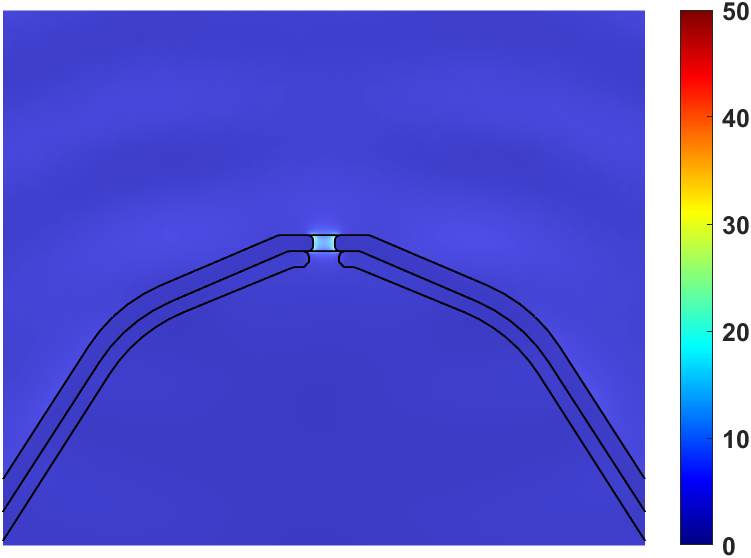 | 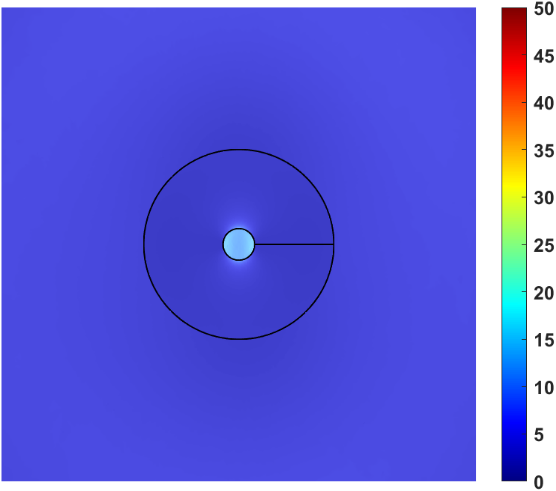 |
| --- | --- |
| 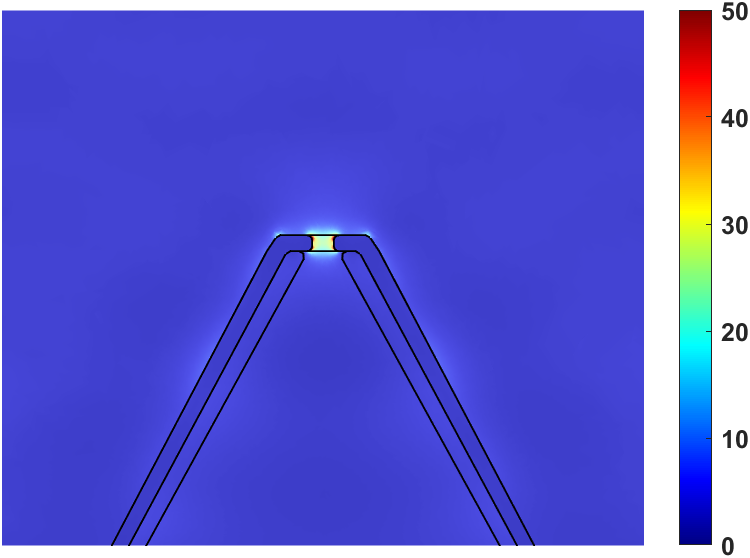 | 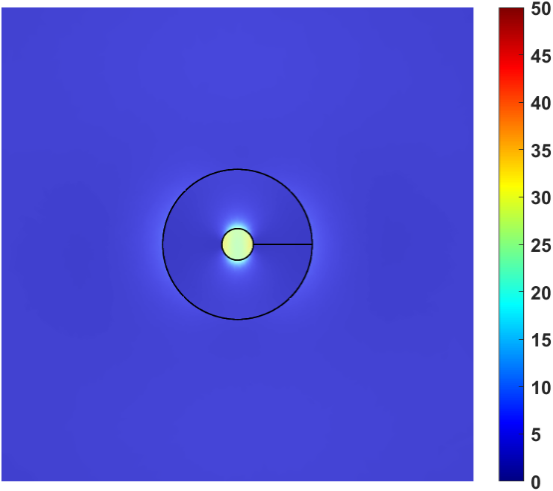 |
| 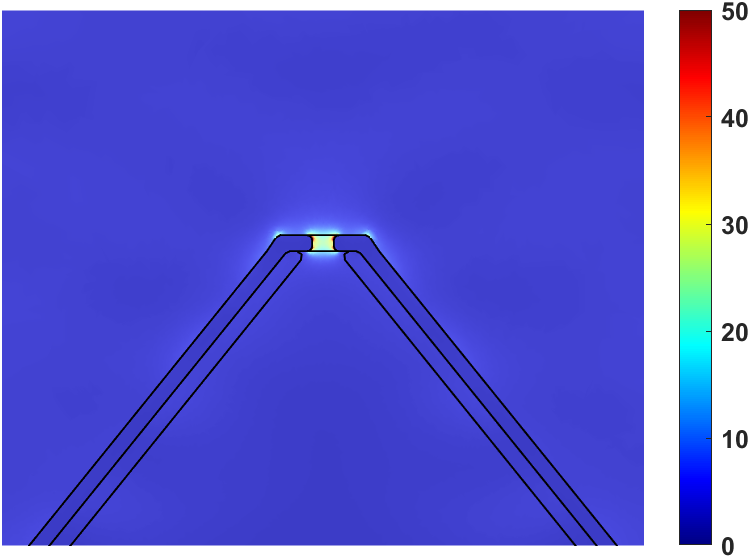 | 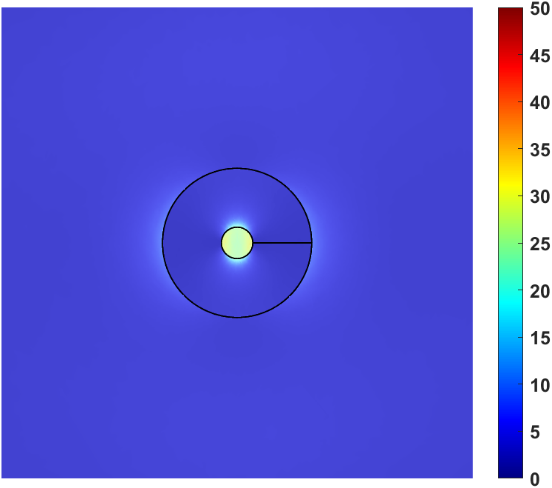 |

Figure S17. Simulated electric field enhancement (∣𝐸∣^2^/∣𝐸_0_∣^2^) at λ = 634 nm for plasmonic nanopores. Cross-sectional and top-view maps of the field intensity for a gold-coated conical nanopore with rounded edges. The simulations were performed at λ = 634 nm, a wavelength selected close to the experimental illumination at 640 nm. The mode produces strong, symmetric confinement around the pore, with maximum intensity within a few nanometers from the metal surface. (c, d) Corresponding maps for a dual-material nanopore combining gold and silicon. The near field is asymmetrically localized toward the metallic side, while the dielectric region shows minimal enhancement at this wavelength.


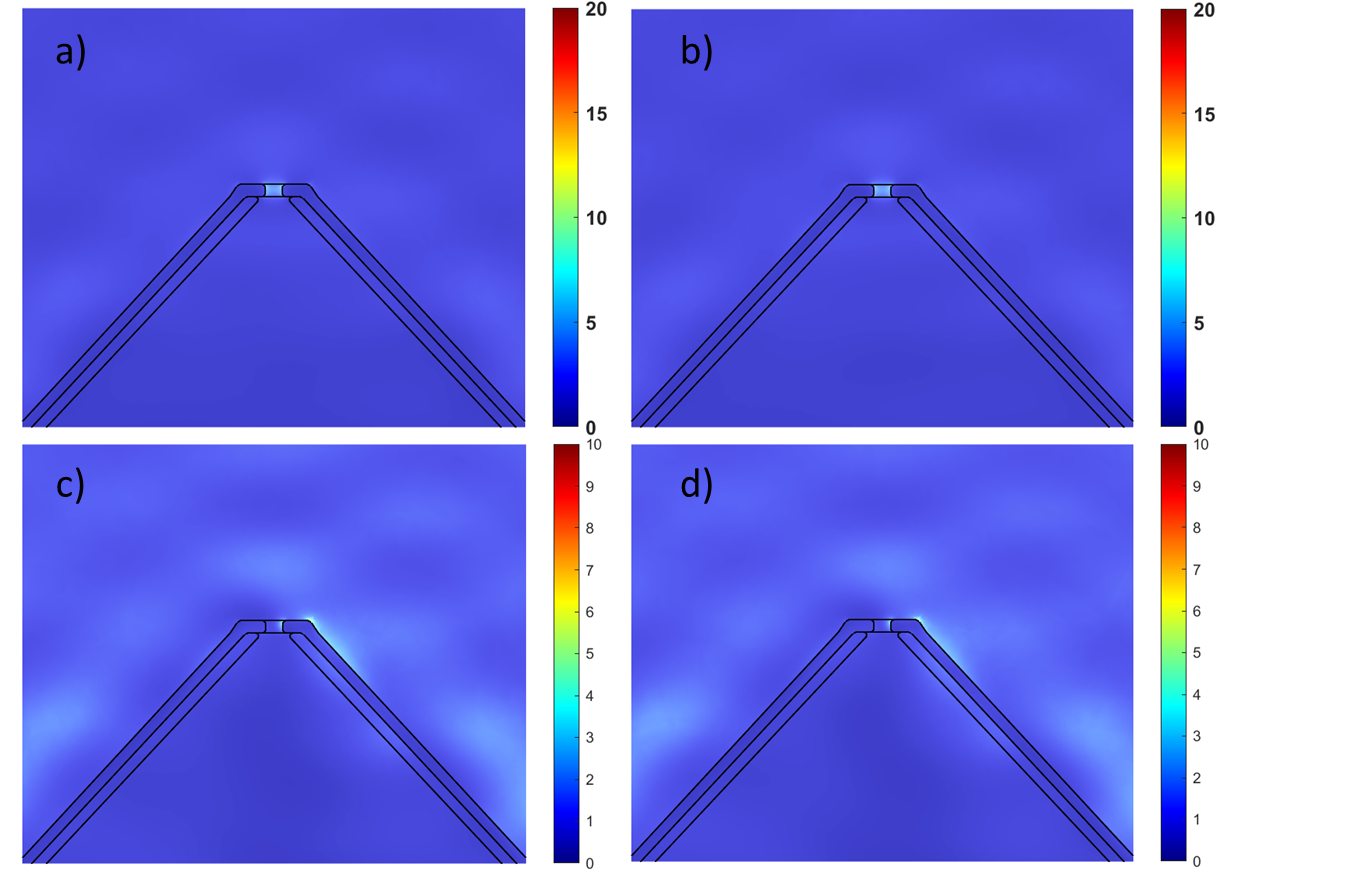


Figure S18. Simulated electric field enhancement (∣𝐸∣^2^/∣𝐸_0_∣^2^) at λ=634 nm for nanopores under circularly polarized excitation. (a,b) Cross-sectional maps for the Au nanopore under left- and right-handed circular polarization, respectively. (c,d) Cross-sectional maps for the dual-material Au/Si nanopore under left- and right-handed circular polarization, respectively. For both geometries, the two handednesses yield the same qualitative field distribution and hotspot localization, indicating that the overall enhancement pattern is not materially affected by the handedness of circular polarization under the present non-chiral nanopore geometry.

# References

(1) Schnitzbauer, J.; Strauss, M. T.; Schlichthaerle, T.; Schueder, F.; Jungmann, R. Super-Resolution Microscopy with DNA-PAINT. *Nat Protoc* 2017, *12* (6), 1198–1228. https://doi.org/10.1038/nprot.2017.024.
